# Amino acid substitution scoring matrices specific to intrinsically disordered regions in proteins

**DOI:** 10.1038/s41598-019-52532-8

**Published:** 2019-11-08

**Authors:** Rakesh Trivedi, Hampapathalu Adimurthy Nagarajaram

**Affiliations:** 1 Laboratory of Computational Biology, Centre for DNA Fingerprinting and Diagnostics, Uppal, Hyderabad, Telangana 500039 India; 2 Graduate School, Manipal Academy of Higher Education, Manipal, Karnataka 576104 India; 3 Department of Systems and Computational Biology, School of Life Sciences, University of Hyderabad, Hyderabad, Telangana 500 046 India; 4 Centre for Modelling, Simulation and Design, University of Hyderabad, Hyderabad, Telangana 500 046 India

**Keywords:** Intrinsically disordered proteins, Protein sequence analyses

## Abstract

An amino acid substitution scoring matrix encapsulates the rates at which various amino acid residues in proteins are substituted by other amino acid residues, over time. Database search methods make use of substitution scoring matrices to identify sequences with homologous relationships. However, widely used substitution scoring matrices, such as BLOSUM series, have been developed using aligned blocks that are mostly devoid of disordered regions in proteins. Hence, these substitution-scoring matrices are mostly inappropriate for homology searches involving proteins enriched with disordered regions as the disordered regions have distinct amino acid compositional bias, and therefore expected to have undergone amino acid substitutions that are distinct from those in the ordered regions. We, therefore, developed a novel series of substitution scoring matrices referred to as *EDSSMat* by exclusively considering the substitution frequencies of amino acids in the disordered regions of the eukaryotic proteins. The newly developed matrices were tested for their ability to detect homologs of proteins enriched with disordered regions by means of SSEARCH tool. The results unequivocally demonstrate that *EDSSMat* matrices detect more number of homologs than the widely used BLOSUM, PAM and other standard matrices, indicating their utility value for homology searches of intrinsically disordered proteins.

## Introduction

A stable three - dimensional native structure has been considered to be the obligatory prerequisite condition for a protein to perform its biological function^1^. However, there are many naturally occurring functional proteins that do not attain stable three - dimensional structures and appear unfolded. Such proteins have been referred to as intrinsically disordered proteins (IDPs). In many cases, instead of the whole protein, only some segments in the protein are disordered, and such peptide segments have been referred to as intrinsically disordered regions (IDRs)^2^. Interestingly, intrinsically disordered regions have been known to endow proteins with functional promiscuity^3^.

Structural disorder is not an uncommon feature among proteins, and the proportion of disorder increases as the complexity of genomes increases from bacteria, archaea to eukaryotes with a sharp increase at the prokaryote/eukaryote boundary^4–6^. About 33% of eukaryotic proteins contain at least one functionally relevant long (>30 residues) intrinsically disordered region in comparison to 2.0% in archaean and 4.2% in eubacterial proteins^7^. It is interesting to note that IDRs often harbour short linear motifs (3–10 amino acid residues) which anchor with their cognate structural domains of other proteins thereby enabling protein-protein physical interactions^8,9^.

Several studies have been reported on comparative analyses of features such as sequence complexity, amino acid compositions and their frequencies, and evolutionary rates^10–13^, which have given rise to a reasonable understanding of the evolution of disordered regions in proteins. The presence of short functional sites, low content of bulky hydrophobic residues and, a high proportion of polar and charged amino acids are a few specific characteristics of the IDRs in proteins^14^. The evolutionary rates of the IDRs are significantly higher than the ordered regions, because of which insertions and deletions appear more frequently in these regions^15,16^.

The distinct compositional bias and higher evolutionary rates of IDRs as compared with the ordered regions together indicate that substitution frequencies of residues in the disordered regions are also distinct from those found in ordered regions. Therefore, the use of scoring matrices developed from ordered regions of proteins for any sequence analyses such as homology searches of IDPs is inappropriate. We, therefore, felt that it is highly essential to develop new substitution matrices appropriate for disordered regions in proteins.

In this work, we have developed substitution matrices appropriate for homology searches involving eukaryotic proteins enriched with IDRs. These matrices were developed using Henikoff’s method^17^ from a curated dataset of alignments of eukaryotic proteins belonging to about 4000 families. The newly developed matrices were evaluated by performing homology searches using SSEARCH tool on a large data set of query proteins (39788338) enriched with different percentage of IDRs, and the overall sensitivity as given by the score Coverage Measure (Q) was calculated. The results indicate that the newly developed disordered specific matrices perform significantly better than the widely used matrices such as BLOSUM^17^, PAM^18^ in their ability to detect homologs for proteins enriched with IDRs, and hence are useful in homology searches involving such proteins.

## Materials and Methods

### Dataset preparation

We considered an exhaustive dataset (referred to as EUMAT dataset) of 4189 eukaryotic protein families comprising of 36498 proteins extracted from uniprot database. All the proteins in this dataset are with the protein existence evidence (PE = 1), and are of minimum sequence length of 100 amino acids. The average length of proteins in these families varies between 100 to 5195 residues, with the median value of 400. Number of members in protein families ranges between 2 to 812, and more than 60% of protein families contain at least 4 proteins.

### Clustering and multiple sequence alignment (MSA) of protein families

Amino acid sequences of proteins in the EUMAT dataset were retrieved from UniProtKB^19,20^. The protein sequences within each family were clustered at various % identity levels (50%, 60%. 62%, 70%, 75%, 80% and 90%), and the proteins that are representative of each cluster (i.e. centroids of clusters) were retrieved using Usearch^21^. Details of the numbers of protein sequences and the families at different % identity level are given in Supplementary Table S1. Sequence alignments of centroids identified at various sequence identity levels in each family were performed using PRANK^22^ with default parameters (gap opening rate = 0.005, gap extension probability = 0.5, number of iterations = 5). We used ‘+F’ variant of PRANK which imposes an insertion pattern in accordance with phylogeny, and avoids overestimation of deletion events^23^.

### Identification of IDRs in proteins and generation of alignment blocks

We identified IDRs by predicting the disordered regions in proteins. Currently there are more than 50 methods available for predicting disordered regions in proteins, and among them IUPred long^24^ has been shown to perform well^25^. We therefore used IUPred long to predict disorders regions in the proteins of EUMAT dataset. Additionally, we also used SSpro^26^ from SCRATCH Protein Predictor package to predict secondary structures in those proteins. We identified an amino acid residue as part of disordered region only if it is predicted to be part of disordered region by IUPred long, and also as part of coil region as predicted by SSpro. This is because the evolution of coil regions is similar to disordered regions^11^. Furthermore, protein sites predicted as intrinsically disordered, and which are also part of secondary structure i.e. coils are generally considered as highly conserved and functionally more relevant residues of proteins^27^. Of all the residues in the complete EUMAT dataset, ~16% were predicted to be in IDRs (Supplementary Table S2). The alignment columns comprising of only disordered annotated residues were separately pooled together to form disordered alignment blocks for every protein family, and this pooling was done for protein alignments corresponding to different sequence identity levels. The details of the number of disordered alignment blocks and amino acid pairs at various sequence identity levels are given in Supplementary Table S3. The columns having gaps or residues with mixed structural states were ignored.

### Compilation of amino acid substitution scoring matrices

Substitution scoring matrices were computed using the scripts developed by us that implement the Henikoff’s method^17^. Briefly, in this method the number of amino acid transitions involving all possible 210 amino acid pairs are counted from blocks, and using these counts, observed and expected probabilities of occurrence of all residue pairs are computed which are further converted into scaled logarithmic values (Log Odd ratios (LOD)). We computed matrices from disordered alignment blocks at different sequence identity levels. Henceforth, the developed matrices are referred to as Eukaryotic Disorder Substitution Scoring Matrix (*EDSSMat*) series of matrices. For all these matrices we further computed their relative entropies (H), expected scores (E) and matrix averages (average of all 210 residue pairs Log Odd Scores) (Table 1).

**Table 1:** Matrix parameters (Matrix average, Expected score (E), and Relative entropy (H)) corresponding to various *EDSSMat* series of matrices.

Matrix Parameters	EDSSMat50	EDSSMat60	EDSSMat62	EDSSMat70	EDSSMat75	EDSSMat80	EDSSMat90
Matrix Average	−0.800	−0.838	−0.828	−0.828	−0.828	−0.838	−0.871
Expected Score (E)	−0.2347	−0.2339	−0.2345	−0.2355	−0.2351	−0.2374	−0.2458
Relative Entropy (H)	0.9099	0.9099	0.9129	0.9159	0.9109	0.9169	0.9459

### Evaluation of performance of matrices for homology detection

In order to detect homologs with varying degree of disorderedness, the EUMAT dataset was divided into three test datasets viz., (a) Less Disordered (LD) (0% to <=20% disorderedness), (b) Moderately Disordered (MD) (>20% to <=40% disorderedness) and (c) Highly Disordered (HD) (>40% disorderedness) datasets. Composition of LD, MD and HD test datasets in terms of the number of proteins and the number of protein families are given in Supplementary Table S4. Distribution of percent disorderedness and identities across LD, MD and HD test datasets are given in Supplementary Figs S1 and S2. Most of proteins in the LD, MD and HD datasets possess a higher degree of sequence divergence, and therefore the substitution frequencies computed from their alignments are expected to give rise to matrices with high sensitivities even when working with highly diverged sequences^28,29^.

We employed SSEARCH from FASTA package (Version: 36.10) to evaluate the utility value of *EDSSMat* matrices with respect to various commonly used search matrices. Among the homology detection tools, SSEARCH has been reported as the most sensitive similarity search method^30,31^.

Furthermore, for the sake of convenience, various scoring matrices were grouped as *Standard*, *Disorder* and *EDSSMat* as detailed in Table 2. The group of matrices referred to as *Standard* includes BLOSUM, PAM, MD^32^ and VTML^33,34^ series of scoring matrices which are routinely used as default matrices in the popular homology search tools such as SSEARCH/FASTA^35^ and BLAST^36^. The second group of matrices referred to as *Disorder* comprises of previously developed disordered region-specific scoring matrices, (Henceforth, matrices developed by Radivojac *et al*.^10^, Brown *et al*.^11^ and Midic *et al*.^12^ will be referred to as DUNMat, Disorder85, Disorder60 and Disorder40 (depending on levels of sequence similarity) and MidicMat respectively). The group *EDSSMat* are the matrices developed in this study.

**Table 2:** Substitution scoring matrices set used in homology search performance evaluation.

Matrix Sets	Algorithm	Matrix Numbers
*EDSSMat* Matrices	EDSSMat	50, 60, 62,70, 75, 80, 90
*Standard* Matrices	BLOSUM	30, 50, 62, 80
	PAM	120, 250
	MD	10, 20, 40
	VTML	10, 20, 40, 80, 120, 160, 200
*Disorder* Matrices	DUNMat	—
	Disorder	40, 60, 85
	MidicMat	—

Exhaustive homology searches were performed using all the matrices with gap opening and gap extension penalties ranging from −5 to −20 and −1 to −3, respectively, and optimum set of gap penalties for each matrix were identified as those which give rise to the maximum number of true homologs. Evaluation of homology search performance of various matrices was performed using the metric called Coverage Measure (Q)^37^ which represents the fraction of correctly found true positive family relations (homologs) when a restricted number of false positives are allowed. As per the convention followed in literature^29,37^, we permitted one false positive for every 100 queries (i.e., the numbers of errors per query (EPQ) = 0.01). As the number of relationships within a protein family varies quadratically with respect to family size, we, implemented a suggestion by Price *et al*.^28^ and used quadratically normalized version of coverage measure (Q_quad_):

1 $${{\rm{Q}}}_{{\rm{quad}}}=\frac{1}{{\rm{S}}}\mathop{\sum }\limits_{{\rm{i}}=1}^{{\rm{S}}}\frac{{{\rm{t}}}_{{\rm{i}}}}{({{\rm{s}}}_{i}^{2}-{{\rm{s}}}_{i})}$$

Here S represents the number of families in database, and *t*_*i*_ the number of true positive relations found for a family *i* which contains *s*_*i*_ sequences. These values (Q_quad_) were computed using the CoverageCalculator tool^29^, a performance optimised reimplementation of PSCE toolkit^28,30,31,38^.

Statistical significance of homology detection by different matrices was evaluated by means of Concerted Bayesian bootstrapping method. This was done to analyse the implications of variations in database composition on the number of homologs detected. Sequence weights of the prior were obtained from the Dirichlet distribution, and quadratic normalisation of the resulting bootstraps was performed as described in previous studies^29^. Prior distributions were generated 500 times.

Z-score statistics was used to measure the statistical significance of the results obtained by various matrices^33^. Z-score calculation for a pair of bootstrap distributions M and P is given as follows:

2 $${{\rm{Z}}}_{{\rm{M}},{\rm{P}}}=\frac{{({\bar{{\rm{Q}}}}_{{\rm{quad}}})}_{{\rm{M}}}-{({\bar{{\rm{Q}}}}_{{\rm{quad}}})}_{{\rm{P}}}}{\sqrt{\frac{{\sigma }_{{\rm{M}}}^{2}+{\sigma }_{{\rm{P}}}^{2}}{{\rm{N}}}}}$$

where, ${({\bar{{\rm{Q}}}}_{{\rm{quad}}})}_{{\rm{M}}}\,$and ${({\bar{{\rm{Q}}}}_{{\rm{quad}}})}_{{\rm{P}}}$; σ^2^_M_ and σ^2^_P_ are the means and variances of bootstrap coverages calculated at EPQ of 0.01 for the matrices M and P respectively. N represents number of bootstrap steps^37^. A value Z ≥ 1.96 is considered significant as Z = 1.96 pertains to 97.5 percentile of distribution^28^.

We also compared the distribution of E-values corresponding to the common homologs detected by all the matrices with an expectation that *EDSSMat* matrices should give rise to better E-values than the other matrices.

## Results and Discussion

### Calculation of substitution matrices and their characterisation

*EDSSMat* matrices at various identity levels were computed by following Henikoff’s method as mentioned in the methods section. Table 1 gives the values of various matrix parameters viz., matrix average, expected score (E) and relative entropy (H). As can be seen from the table, averages of LOD scores and expected score (E) for all the *EDSSMat* matrices are negative. This means that mismatches in the *EDSSMat* are, in general highly, penalised thereby ensuring that these matrices when used during alignments produce high scoring local alignments which are of biological relevance. High scoring local alignments help in better understanding of alignment statistics^39^. Expected score (E) should be negative for a substitution matrix, if the alignment scores have to be used for statistical tests^40,41^. The Smith-Waterman algorithm which rigorously calculates local sequence alignments requires scoring matrices that produce negative average similarity scores for random sequences^42^. If the matrix average or expected score is positive, alignments will extend to the ends of the sequences, and become global, rather than local^43^. Relative entropy (H) of a matrix describes the difference between target (or observed) distribution of pair frequencies with respect to background (or expected) distribution, and positive entropies values of *EDSSMat* matrices reflects that both these distributions are quite distinguishable.

**Figure 1:**
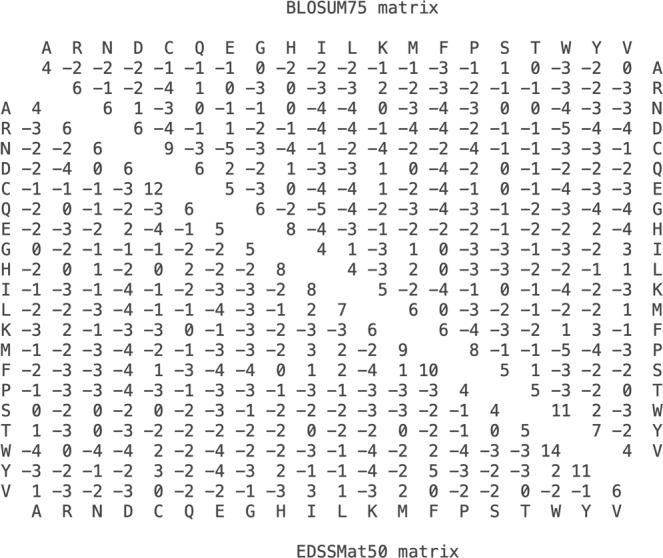
Relative entropy-dependent comparison of LOD scores of BLOSUM and *EDSSMat* series of matrices. Upper half diagonal represents BLOSUM75 (H ~ 0.9 bits) and lower half diagonal represents EDSSMat75 (H = 0.909 bits) matrix values.

In order to understand the significance of matrix values, we compared log odd scores of EDSSMat50 (H = 0.909 bits) and commonly used BLOSUM series matrices (BLOSUM75; H ~ 0.9 bits) in relative entropy-dependent manner^40^. As shown in Fig. 1, in EDSSMat50 matrix identical amino acid matches are assigned higher scores in comparison to BLOSUM75 (EE, KK, FF, II, CC etc.). It is interesting to note that in contrary to BLOSUM matrices that tends to penalize matching of non-identical residues, *EDSSMat* tends to assign higher scores (DE, FY, IM, HQ etc.) or smaller penalties (AF, GI, HI, LP etc.) to the matching of non-identical residues in disordered regions, where such mismatches are more likely to occur spontaneously due to higher evolutionary rate.

### Relative entropy-dependent comparison of homology search performances

Performance of a substitution matrix can be measured as the number of true homologs detected (sensitivity) as well as its ability not to detect non-homologs (specificity) during database searches by a search tool. We assessed homology search performance of our *EDSSMat* series of matrices in comparison to the *Standard* and *Disorder* group of matrices on LD, MD and HD test datasets using a number of gap penalties as mentioned in the methods section. Optimum gap penalties (which gave rise to the highest number of true homologs) for various matrices on different test datasets along with their coverage values (Q_quad_) at EPQ = 0.01 are given in Supplementary Table S5.

A fair comparison of homology search performance between matrices can be achieved only if they have comparable relative entropies^40^, as relative entropy explains the divergence of observed substitution events and independent evolution captured within a substitution matrix. The relative entropy of *EDSSMat* series ranges between 0.9099 bits (EDSSMat50) and 0.9459 bits (EDSSMat90) (Table 1). Of all the search matrices, only VTML120 (H = 0.9382 bits) have comparable entropy to *EDSSMat* series (EDSSMat90; H = 0.9459 bits) matrix, hence they can be directly compared. The other matrices in the *Standard* and *Disorder* groups could not be compared with *EDSSMat* matrices as their relative entropies are either not comparable or have not been reported.

Coverage values achieved using best performing gap parameters at 0.01 errors per query (EPQ) by EDSSMat90 and VTML120 on LD, MD and HD test datasets, and their performance differences statistical significance computed through Z-score statistics using Concerted Bayesian bootstrap are given in Table 3. It is clearly evident from coverage values and Z-scores that EDSSMat90 performance is significantly better than VTML120 on all three LD, MD and HD test datasets. Also, the homology search performance of both EDSSMat90 and VTML120 grows with increasing percent disorderedness of datasets being tested, i.e. coverage is least on LD test dataset whereas maximum on HD test dataset. These findings suggest that EDSSMat90 is a better choice among the matrices with equivalent relative entropies while performing homology searches for proteins with varying degree of disorderedness.

**Table 3:** Relative entropy-dependent comparison of EDSSMat90 (0.9459 bits) and VTML120 (0.9382 bits) matrices for homology detection on all three test datasets.

Dataset	${({\bar{{\bf{Q}}}}_{{\bf{quad}}})}_{{\bf{M}}}$ [EDSSMat90 Coverage at 0.01 EPQ]	${({\bar{{\bf{Q}}}}_{{\bf{quad}}})}_{{\bf{P}}}$ [VTML120 Coverage at 0.01 EPQ]	Z- Score
Less Disordered (LD)	0.3255	0.3018	461.90
Moderately Disordered (MD)	0.5051	0.4599	190.85
Highly Disordered (HD)	0.6604	0.6392	95.253

Ability of matrices to detect homologs are tabulated as Coverage measure (Q_quad_), and significance of their coverage differences are reported as Z-score values.

### Relative entropy-independent comparison of homology search performances

**Figure 2:**
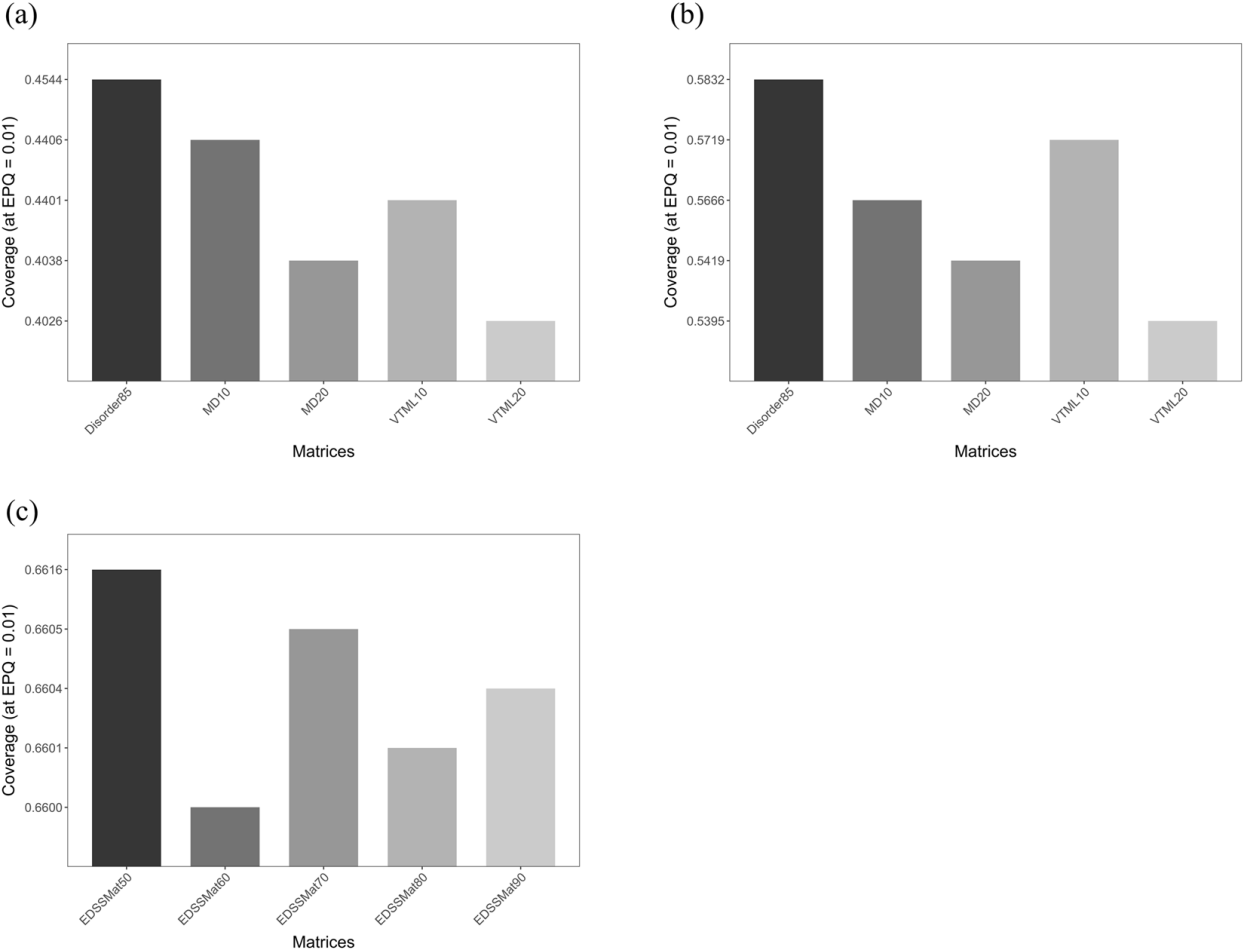
Relative entropy-independent comparison of top 5 search matrices for homology detection using three test datasets: (**a**) Less Disordered (LD); (**b**) Moderately Disordered (MD); and (**c**) Highly Disordered (HD). Quadratically normalised coverage measure (Q_quad_) at 0.01 errors per query (EPQ) on y axis reports the fraction of true positive family relations at a restricted number of false positives. Height of a bar in the figure represents coverage (Q_quad_) achieved by a matrix. All *EDSSMat* series of matrices achieved higher coverage values (Q_quad_) than other comparing matrices on HD test dataset. On MD and LD test datasets, along with Disorder85, lower numbered MD and VTML search matrices are the best performers. Differences in coverage measure are also statistically significant as |Z| ≥ 1.96 (Supplementary Tables 6–8).

While the entropy-dependent evaluation compares substitution matrices based on their general compositional properties, an entropy-independent comparison focuses on best performing substitution matrices in a given test setting. Therefore, we compared homology search performances of matrices in entropy-independent manner, and the results of top five performing search matrices on LD, MD and HD test datasets are shown in Fig. 2a–c respectively. On both less and moderately disordered test sets (i.e. LD and MD datasets), matrices with higher information content i.e. VTML10 (H = 3.462 bits) and VTML20 (H = 2.921 bits), and modern PAM-based matrices MD10 and MD20 along with *Disorder* search matrix Disorder85 perform well as compared to the others (Supplementary Tables S6 and S7). *EDSSMat* series matrices (EDSSMat50 (H = 0.6616 bits), EDSSMat70 (H = 0.6605 bits), EDSSMat90 (H = 0.6604 bits), EDSSMat80 (H = 0.6601 bits) and EDSSMat60 (H = 0.6600 bits)) are the five best performing scoring matrices on highly disordered (HD) test dataset. In general, coverage attained at 0.01 EPQ on HD test dataset by *EDSSMat* series matrices is higher than all search matrices used in the study (Supplementary Table S5), and also differences in their coverage values are statistically significant (Supplementary Table S8).

In addition, we also performed comparative homology search performance evaluation of the matrices by taking top 20 most populated protein families from each of the test dataset [Highly Disordered (HD), Moderately Disordered (MD) and Less Disordered (LD)]. Similar to our previous results of all vs all comparison, we found that our *EDSSMat* set of matrices are the best performers in homologs searches on highly disordered (HD) test dataset (Supplementary Fig. S3).

Furthermore, *EDSSMat* matrices outperformed most commonly used BLOSUM series of matrices on all three test datasets. Although difference between their coverage values on LD test dataset is only subtle but still statistically significant. Both PAM120 and PAM250 matrices exhibit similar performances like BLOSUM series, and are also outperformed by *EDSSMat* search matrices on all three test datasets. The remarkable observation we have made is that *EDSSMat* matrices perform better than *Disorder* set matrices (DUNMat, Disorder85, Disorder60. Disorder40 and MidicMat) on all three datasets, except Disorder85 and Disorder60 matrices on MD and LD test datasets, and DUNMat on MD test dataset. MidicMat, the only disordered matrix with practical application till date^44^ was always found to be underperforming in comparison to *EDSSMat* series of matrices on all the three test datasets (Supplementary Table S5).

Better performance of *EDSSMat* as compared to MidicMat can be attributed to the following: (a) size and heterogeneity of dataset used for matrix compilation, (b) quality of alignments (c) method used for computation of substitution scores, and also (d) prediction of disordered regions. This is well reflected in the LOD scores. In contrast to MidicMat that tends to assign higher scores to substitutions involving polar/charged and hydrophobic residues, *EDSSMat* matrices tends to penalize such substitutions (AD, SV, LT, AN, AR, PQ etc.), as disordered regions are enriched with polar/charged residues. Hence it is clearly evident from relative entropy-independent evaluation of homology search performance of the *EDSSMat*, *Standard* and *Disorder* search matrices that the *EDSSMat* series is best homolog search matrices for proteins harboring higher degree of disorderedness.

### Comparison of E-value distributions obtained for the homologs commonly detected by scoring matrices during homology searches

**Figure 3:**
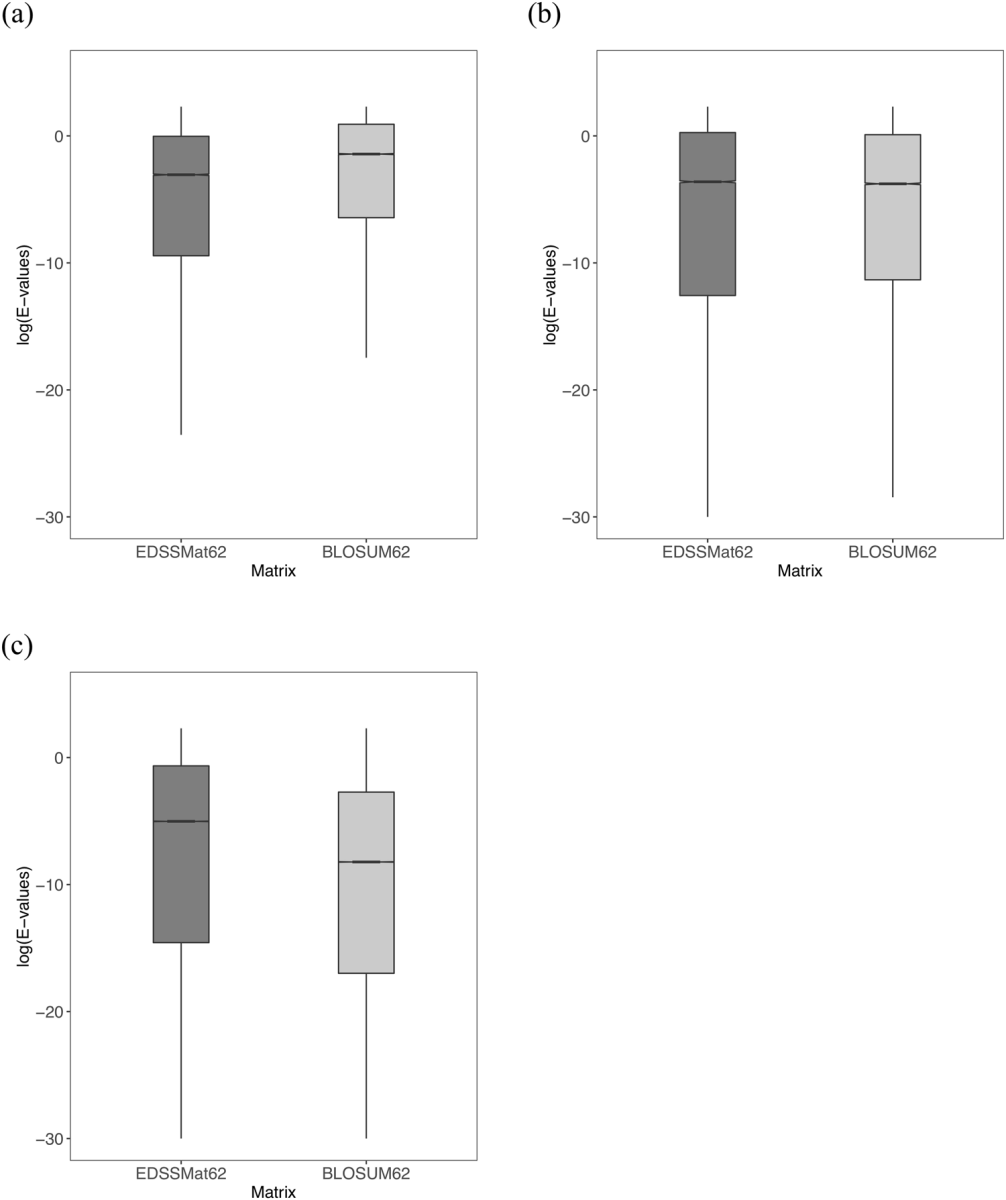
Common homologs E-values distribution of BLOSUM and *EDSSMat* series of matrices. For representative purpose comparison of log10(E-values) distributions of common homologs of BLOSUM62 and EDSSMat62 on three different test datasets: **(a)** Highly Disordered (HD); **(b)** Moderately Disordered (MD); and **(c)** Less Disordered (LD) is shown here. EDSSMat62 matrix achieved lower E-values on dataset comprised of highly disordered proteins i.e. HD test dataset, whereas BLOSUM62 attained lower E-values on dataset enriched with ordered regions. Difference in E-values distributions are statistically significant (wilcoxon test, p-value is < 2.2e-16).

**Figure 4:**
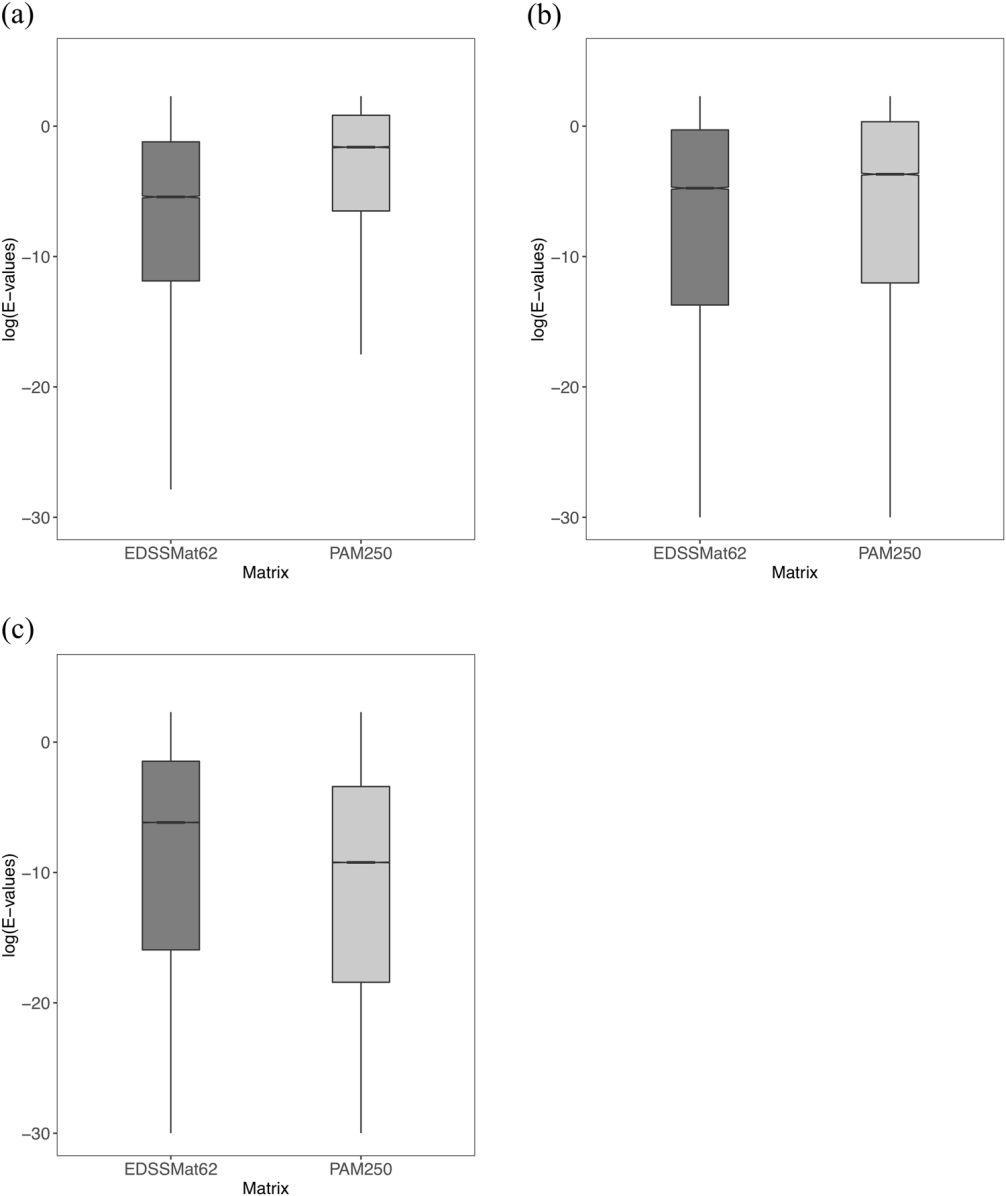
Common homologs E-values distribution of PAM and *EDSSMat* series of matrices. For representative purpose comparison of log10(E-values) distributions of common homologs of PAM250 and EDSSMat62 on three different test datasets: (**a**) Highly Disordered (HD); (**b**) Moderately Disordered (MD); and (**c**) Less Disordered (LD) is shown here. EDSSMat62 matrix achieved lower E-values on dataset comprised of highly disordered proteins i.e. HD test dataset, whereas PAM250 attained lower E-values on dataset enriched with ordered regions. Difference in E-values distributions are statistically significant (wilcoxon test, p-value is < 2.2e-16).

**Figure 5:**
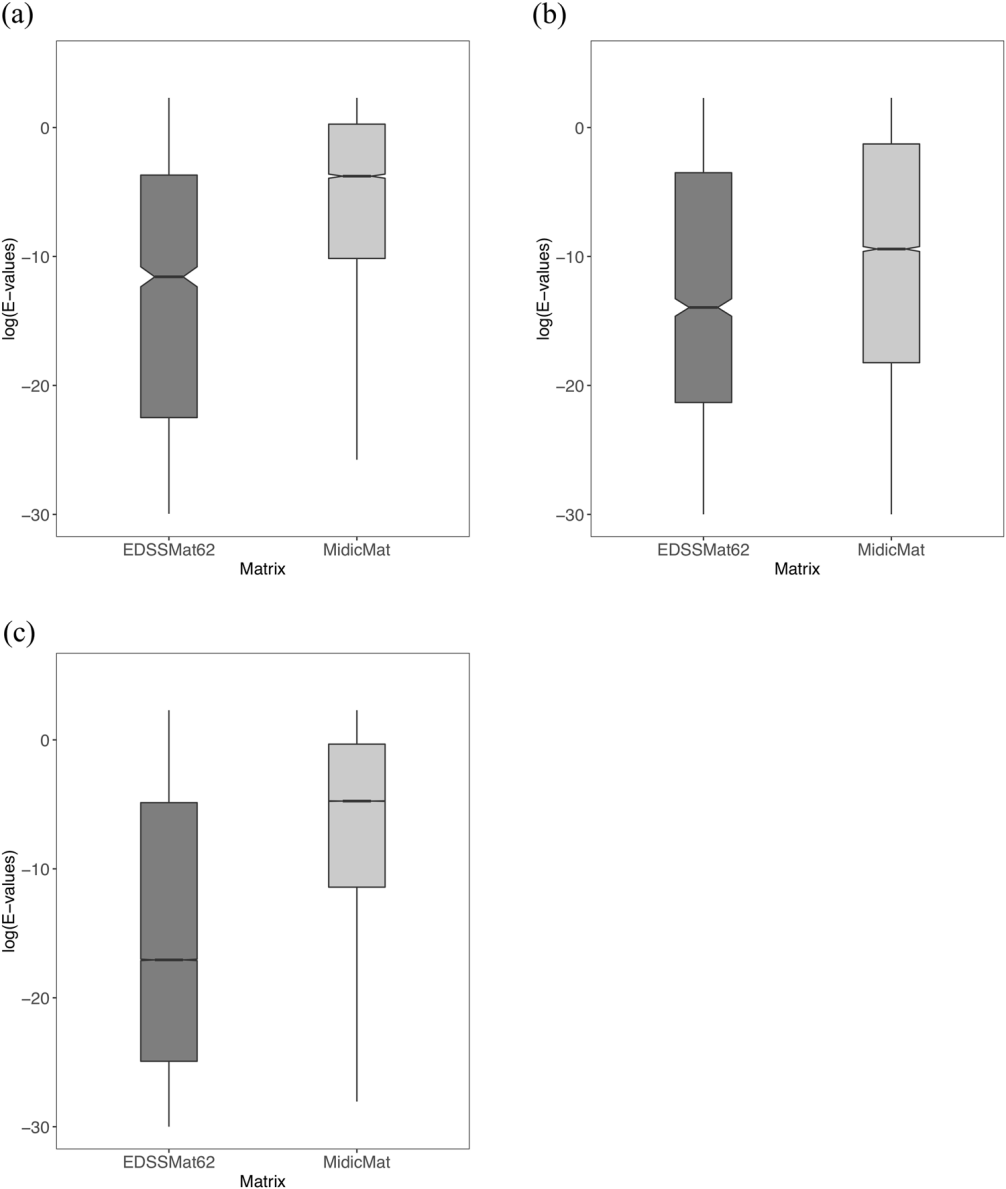
Common homologs E-values distribution of MidicMat and *EDSSMat* series of matrices. For representative purpose comparison of log10(E-values) distributions of common homologs of MidicMat and EDSSMat62 on three different test datasets: **(a)** Highly Disordered (HD); **(b)** Moderately Disordered (MD); and **(c)** Less Disordered (LD) is shown here. Irrespective of the disorder content of the test datasets, EDSSMat62 matrix always achieved lower E-values than MidicMat, and these differences in E-values distributions are also statistically significant (wilcoxon test, p-value is < 2.2e-16).

In homology searches, E-values form the best metric for the statistical significance of a hit for a given query sequence. The lower the E-value, the more significant the substitution scores for a query - hit pair. Therefore, we studied distribution of E-values corresponding to the common homologs detected by *EDSSMat* series matrices with respect to each of the *Standard* and *Disorder* set matrix in pairwise manner. All *EDSSMat* matrices achieved significantly lower E-values on highly disordered (HD) test dataset in comparison to BLOSUM and PAM series of matrices (Figs 3a and 4a). Even with respect to most of the VTML, MD and *Disorder* set of matrices, *EDSSMat* matrices attained lower E-values on HD test dataset. However, there are fewer incidences when E-values of *EDSSMat* matrices are either marginally high or comparable to that of *Standard* (VTML10, MD10 and MD20) and *Disorder* set (Disorder40 and Disorder85) of matrices on disordered region enriched HD protein test dataset (Supplementary Figs S4 to S21). Differences in E-values of *EDSSMat* matrices with respect to BLOSUM, PAM (Figs 3b and 4b) and DUNMat matrices become less prominent as the disorder content decreases from HD to MD test dataset, and these matrices achieved lower E-values in comparison to *EDSSMat* matrices on common set of homologs on LD test dataset (Figs 3c and 4c). Also, *EDSSMat* matrices scored lower E-values than MD matrices, lower numbered VTML (VTML10, VTML20 and VTML40), MidicMat, Disorder40, Disorder60 and Disorder85 search matrices on both moderately (MD) and less disordered (LD) test datasets (Supplementary Figs S4 to S21). These observations on common homologs clearly indicate that *EDSSMat* series of matrices offer better discrimination and detection of homologs for queries enriched with disordered regions than the other matrices by attaining lower E-values. Of course needless to mention, BLOSUM and PAM are the best for queries enriched with ordered regions. Surprisingly, MidicMat, the only disordered matrix with practical application till date^44^ is completely outperformed by all *EDSSMat* matrices on all three test datasets (Fig. 5a–c). The difference in distribution of E-values for all pairs of search matrices on LD, MD and HD test datasets are statistically significant (wilcoxon test, p-value is < 2.2e-16).

**Figure 6:**
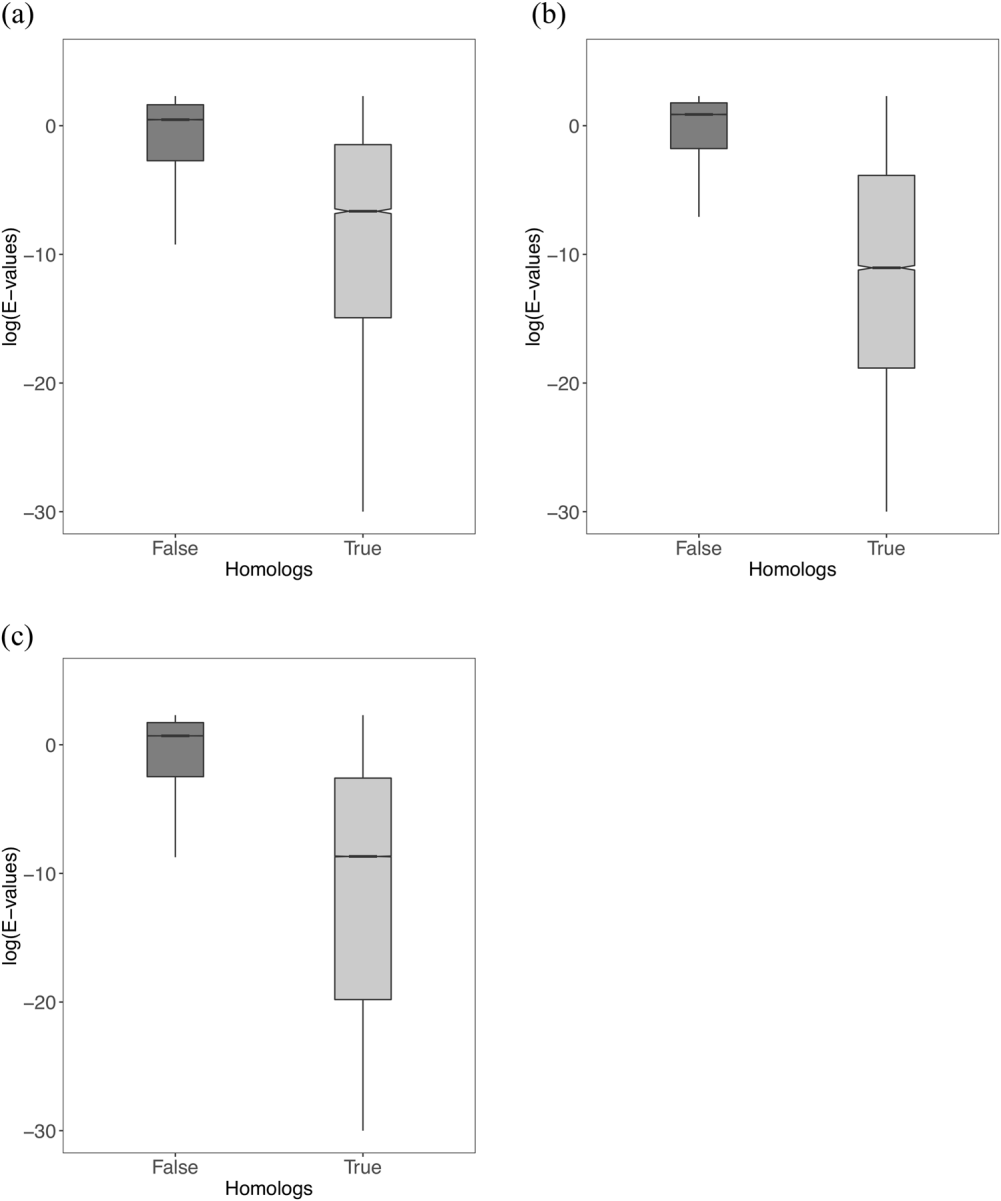
True and False homologs E-values distribution of *EDSSMat* series of matrices. For representative purpose comparison of True and False homologs E-values of EDSSMat62 matrix in SSEARCH assisted homolog searches on three different protein test datasets: (**a**) Highly Disordered (HD); (**b**) Moderately Disordered (MD); and (**c**) Less Disordered (LD) is shown here. True homologs always achieved lower E-values than False homologs, and these differences in E-values distributions are also statistically significant (wilcoxon test, p-value is < 2.2e-16).

We also compared E-values of true positives and false positives obtained during homology searches involving *EDSSMat* matrices on all three (LD, MD and HD) test datasets. It was observed that false positives are associated with higher E-values in comparison to true homologs and the difference is statistically significant (wilcoxon test, p-value is < 2.2e-16) (Fig. 6a–c).

### An example for homolog search using *EDSSMat* matrices

As demonstrated in the previous sections, *EDSSMat* matrices emerge as the best matrices for homology searches involving the proteins enriched with IDRs on a test dataset. We further investigated the usefulness of *EDSSMat* in a general setting where homology searches for an IDP is carried out using a universal dataset. For this we considered a query sequence (uniprot id: O35314) from the chromogranin/secretogranin protein family whose members are known to play essential roles in regulated secretory pathways^45^. SSEARCH based homology searches were carried out for the query sequence against the entire Uniprot Knowledgebase (UniprotKB). Homologs detected for various matrices viz., BLOSUM62, PAM250, MidicMat and EDSSMat62 matrices are given in Table 4.

**Table 4:** Homologs identified by BLOSUM62, PAM250, MidicMat and EDSSMat62 matrices at optimum parameters using uniprot entry O35314 from highly disordered (HD) test dataset as query sequence against Uniprot Knowledgebase (UniprotKB) database.

Search Matrices	Uniprot id of homologs Identified for Query sequence (O35314)	Percent Identity between Query sequence (O35314) and hit	E-values
BLOSUM62	P16014	85.1	0
	P05060	64.7	1.1e–154
	Q9GLG4	54.9	5.7e–111
	P23389	54.3	3.2e–103
PAM250	P16014	85.1	1.3e–146
	P05060	64.7	3.1e–105
	Q9GLG4	54.9	2e–78
	P23389	54.3	1.2e–71
MidicMat	P16014	85.1	0.042
	P05060	64.7	5.2
EDSSMat62	P16014	85.1	0
	P05060	64.7	0
	Q9GLG4	54.9	1.5e–191
	P23389	54.3	4e–182
	**P10645**	22.9	0.93
	**P04404**	21.1	2.2
	**P05059**	20.7	2.9

Additional homologs identified by EDSSMat62 matrix are highlighted in bold.

While BLOSUM62, PAM250 and MidicMat matrices were able to identify some of the close homologs, they failed to identify distant homologs. Only EDSSMat62 was able to identify both close and distant homologs of query sequence (O35314). This clearly shows the utility value of the EDSSMat62 for homology searches of proteins enriched with disordered regions. One need not emphasise the importance of identification of remote homologies especially in the case of families with IDPs.

## Conclusion

In this work, we presented development and evaluation of amino acid substitution matrices, referred to as *EDSSMat* series of matrices, that encapsulate amino acid substitution frequencies in the disordered regions in eukaryotic proteins. In order to develop these matrices we compiled a large dataset of proteins harboring disordered regions; we used double prediction methods IUPred long and SSpro for identifying residues in the disordered regions, and compiled the matrices from aligned disordered blocks using a rigorous Henikoff’s method^17^. It has been shown that these matrices give rise to homology detections with better sensitivities as compared to those routinely used scoring matrices (BLOSUM, PAM, MD and VTML), and also with respect to other previously developed disordered region-specific matrices (DUNMat, Disorder85, Disorder60, Disorder40 and MidicMat) for proteins harboring disordered regions. In fact it was observed that the sensitivity for homology detection (as measured by Q_quad_ values at 0.01 EQP) increases as the disorder content in the query sequence increases. Even in comparisons of E-values distributions of common homologs, our *EDSSMat* series of matrices achieved significantly lower E-values than conventional matrices on sequences enriched with disorderedness. These results unequivocally show that our matrices outperform the widely used BLOSUM and PAM in their ability to detect homologs for proteins with higher degree of disordered regions. These matrices, therefore, will help further studies on evolution and functional characterisation of disordered regions in proteins.

However, we were not able to judge our matrices for their ability to produce accurate sequence alignments for IDPs. This is because of lack of gold standard alignment datasets (structure-based sequence alignments) in the case of protein families with IDPs as members.

## Supplementary information

Supplementary_Information

## Data Availability

EUMAT dataset, three test datasets [Less Disordered (LD), Moderately Disordered (MD) and Highly Disordered (HD)] and *EDSSMat* series of matrices are available at http://www.cdfd.org.in/labpages/computational_biology_project11.html as well as on http://doscb.uohyd.ac.in/han/datasets.php?f_key=LS8992F.
