# Supplementary material for: Amino acid substitution scoring matrices specific to intrinsically disordered regions in proteins
Source: Sci Rep. 2019 Nov 8;9:16380. doi: 10.1038/s41598-019-52532-8 (PMC6841959; doi:10.1038/s41598-019-52532-8)
Supplement: Supplementary file 1 — Supplementary_Information [file 41598_2019_52532_MOESM1_ESM.pdf]

Amino acid substitution scoring matrices specific to intrinsically disordered regions in proteins

Rakesh Trivedi [1,2] and Hampapathalu Adimurthy Nagarajaram [3,4,*]

[1] Laboratory of Computational Biology, Centre for DNA Fingerprinting and Diagnostics, Uppal, Hyderabad, Telangana, 500039, India

[2] Graduate School, Manipal Academy of Higher Education, Manipal, Karnataka, 576104, India

[3] Department of Systems and Computational Biology, School of Life Sciences, University of Hyderabad, Hyderabad, Telangana, 500 046, India

[4] Centre for Modelling, Simulation and Design, University of Hyderabad, Hyderabad, Telangana, 500 046, India

Corresponding author : Prof. Hampapathalu Adimurthy Nagarajaram, Laboratory of Computational Biology, Department of Systems and Computational Biology, University of Hyderabad (UoH), Hyderabad, Telangana, 500046, India; Tel: +91 40 23134561; Email: hansl@uohyd.ac.in.

Supplementary Table S1. Description of total number of protein sequences and families after clustering at various % identity threshold.

Clustering Percentage	Total Number of Proteins	Number of protein families with multiple members after clustering	Number of protein families with single member after clustering
**0**	36498	4189	0
**50**	25111	3859	330
**60**	25633	3878	311
**62**	25768	3880	309
**70**	26323	3894	295
**75**	26790	3915	274
**80**	27467	3942	247
**90**	29780	4040	149

Supplementary Table S2. Composition of EUMAT dataset with respect to disordered residues. Row1 residues with prediction results (IUPred = Disorder and SSPro = Coil) were considered for disordered regions specific matrix compilation.

IUPred Prediction	SSpro Prediction	Number of residues	Percentage of Predictions
Disorder	Coil	2925100	15.938%
Order	Helix/Sheet	8218659	44.781%
Disorder	Helix/Sheet	776349	4.230%
Order	Coil	6432510	35.049%
		Total = 18352618	

Supplementary Table S3. Number of disorder blocks and amino acid pairs contributing to compilation of substitution matrices at different of % identity levels.

	**% Clustering**						
	50	60	62	70	75	80	90
**Number of Blocks**	1859	1865	1868	1862	1869	1881	1949
**Amino Acid Pairs**	706174	704244	705296	699731	709765	709011	763787

Supplementary Table S4. Detailed description of various EUMAT dataset derived test sets (LD, MD & HD) with respect to disorder percentage, total number of proteins and protein families which is used to test homology search performance of various scoring matrices.

**Dataset**	**Dataset Protein Disorder Percentage**	**Total number of protein**	**Total number of proteins families**
Less Disordered (LD)	0% to <= 20%	27832	3352
Moderately Disordered (MD)	>20% to <= 40%	5029	1460
Highly Disordered (HD)	>40 %	3637	938

Supplementary Table S5. Description of optimum gap parameters and maximum coverage achieved by *Standard, Disorder* and *EDSSMat* series search matrices on three different test datasets: Less Disordered (LD), Moderately Disordered (MD) and Highly Disordered (HD).

Matrix	Less Disordered (LD)			Moderately Disordered (MD)			Highly Disordered (HD)		
	Gap Open	Gap Extension	Max Coverage	Gap Open	Gap Extension	Max Coverage	Gap Open	Gap Extension	Max Coverage
**BLOSUM30**	-18	-1	0.3060	-20	-1	0.4957	-18	-3	0.6480
**BLOSUM50**	-10	-1	0.3067	-12	-1	0.4731	-11	-2	0.6338
**BLOSUM62**	-7	-1	0.2908	-7	-1	0.4644	-14	-3	0.6260
**BLOSUM80**	-6	-1	0.3113	-6	-1	0.4665	-10	-3	0.6314
**PAM120**	-6	-1	0.3118	-6	-1	0.4696	-7	-1	0.6304
**PAM250**	-19	-1	0.2666	-12	-1	0.4557	-19	-3	0.6427
**MD10**	-20	-3	0.4403	-19	-2	0.5666	-18	-3	0.6057
**MD20**	-20	-3	0.4038	-17	-3	0.5418	-20	-1	0.6101
**MD40**	-10	-1	0.3611	-10	-1	0.5223	-20	-3	0.6214
**VTML10**	-20	-2	0.4401	-15	-1	0.5719	-8	-1	0.6098
**VTML20**	-20	-3	0.4026	-11	-2	0.5395	-13	-2	0.6125
**VTML40**	-16	-1	0.3409	-6	-1	0.4977	-18	-3	0.6245
**VTML80**	-6	-1	0.3276	-6	-1	0.4914	-17	-3	0.6324
**VTML120**	-6	-1	0.3018	-7	-1	0.4599	-13	-3	0.6362
**VTML160**	-5	-3	0.2825	-12	-1	0.4746	-11	-2	0.6333
**VTML200**	-5	-3	0.2891	-12	-1	0.4779	-9	-3	0.6341
**DUNMat**	-6	-1	0.2844	-6	-1	0.5183	-16	-2	0.6406
**MidicMat**	-20	-3	0.1047	-20	-3	0.4685	-20	-3	0.4432
**Disorder40**	-20	-1	0.2794	-7	-1	0.4993	-7	-1	0.6463
**Disorder60**	-20	-1	0.3528	-16	-1	0.5172	-11	-2	0.6371
**Disorder85**	-20	-1	0.4544	-16	-1	0.5832	-7	-2	0.6114
**EDSSMat50**	-8	-1	0.3187	-6	-2	0.5014	-18	-2	0.6616
**EDSSMat60**	-7	-1	0.3145	-6	-2	0.4971	-14	-3	0.6600
**EDSSMat62**	-8	-1	0.3191	-5	-2	0.5059	-19	-2	0.6594
**EDSSMat70**	-7	-1	0.3211	-5	-2	0.5101	-19	-2	0.6605
**EDSSMat75**	-8	-1	0.3184	-5	-2	0.5037	-19	-2	0.6597
**EDSSMat80**	-7	-1	0.3202	-5	-2	0.5032	-15	-3	0.6601
**EDSSMat90**	-7	-1	0.3255	-5	-2	0.5051	-19	-2	0.6604

Supplementary Table S6. Z-score values for the comparison between five best performing search matrices (Disorder85, MD10, VTML10, MD20 and VTML20) and rest of *Standard, Disorder* and *EDSSMat* search matrices on less disordered (LD) test dataset. Z-scores with |Z| ≥ 1.96 corresponds to > 95% confidence interval and hence significant.

vs	**Disorder85**	**MD10**	**VTML10**	**MD20**	**VTML20**
**BLOSUM30**	1083.59	982.63	996.35	747.38	732.36
**BLOSUM50**	1066.23	966.43	979.49	732.82	718.17
**BLOSUM62**	1219.53	1116.41	1133.35	883.18	866.58
**BLOSUM80**	1010.12	912.51	924.00	681.17	667.36
**PAM120**	1008.17	910.40	921.90	678.63	664.81
**PAM250**	1373.11	1271.95	1290.96	1049.80	1032.40
**MD10**	90.86	—	-3.61	-250.76	-257.47
**MD20**	345.03	250.76	250.95	—	-8.59
**MD40**	647.40	551.59	556.74	309.12	298.25
**VTML10**	95.80	3.61	—	-250.95	-257.74
**VTML20**	351.15	257.47	257.74	8.59	—
**VTML40**	794.97	698.14	705.86	459.36	447.27
**VTML80**	895.70	798.01	807.59	561.55	548.58
**VTML120**	1096.63	997.23	1010.71	765.85	751.04
**VTML160**	1255.07	1154.08	1170.90	926.73	910.32
**VTML200**	1202.31	1101.68	1117.43	872.61	856.70
**DUNMat**	1198.04	1100.55	1115.16	877.59	862.40
**MidicMat**	2916.98	2801.30	2863.23	2648.70	2610.59
**Disorder40**	1278.15	1177.13	1194.37	950.79	934.19
**Disorder60**	694.02	599.58	605.28	362.47	351.43
**Disorder85**	—	-90.86	-95.80	-345.03	-351.15
**EDSSMat50**	966.78	868.27	879.26	633.05	619.41
**EDSSMat60**	986.55	889.04	900.11	656.92	643.30
**EDSSMat62**	946.58	849.87	860.07	617.82	604.64
**EDSSMat70**	948.00	849.65	860.28	613.98	600.51
**EDSSMat75**	975.28	876.16	887.45	639.95	626.13
**EDSSMat80**	945.16	847.81	858.15	614.26	600.98
**EDSSMat90**	894.94	798.96	808.15	566.40	553.71

Supplementary Table S7. Z-score values for the comparison between five best performing search matrices (Disorder85, VTML10, MD10, MD20 and VTML20) and rest of *Standard, Disorder* and *EDSSMat* search matrices on moderately disordered (MD) test dataset. Z-scores with |Z| ≥ 1.96 corresponds to > 95% confidence interval and hence significant.

vs	**Disorder85**	**VTML10**	**MD10**	**MD20**	**VTML20**
**BLOSUM30**	372.05	319.12	294.96	199.60	175.26
**BLOSUM50**	471.05	416.24	391.23	299.09	267.23
**BLOSUM62**	518.48	461.72	435.84	343.95	307.72
**BLOSUM80**	502.74	447.02	421.60	330.13	295.64
**PAM120**	493.88	437.77	412.19	319.66	285.49
**PAM250**	553.19	496.25	470.27	380.20	341.56
**MD10**	68.68	21.53	—	-104.08	-106.47
**MD20**	177.84	127.18	104.08	—	-9.95
**MD40**	260.86	209.24	185.68	85.42	69.23
**VTML10**	47.16	—	-21.53	-127.18	-127.91
**VTML20**	174.77	127.91	106.47	9.95	—
**VTML40**	359.25	307.21	283.45	188.74	165.62
**VTML80**	384.28	332.04	308.18	214.67	189.82
**VTML120**	524.00	468.79	443.59	354.01	318.50
**VTML160**	464.60	409.94	384.99	292.69	261.34
**VTML200**	444.51	390.95	366.48	274.53	245.11
**DUNMat**	267.89	218.12	195.38	98.97	82.58
**MidicMat**	480.07	426.48	401.99	312.06	280.39
**Disorder40**	367.87	313.27	288.38	190.11	165.42
**Disorder60**	270.34	220.92	198.33	102.70	86.22
**Disorder85**	—	-47.16	-68.68	-177.84	-174.77
**EDSSMat50**	338.75	287.80	264.51	170.34	148.96
**EDSSMat60**	371.15	317.46	292.97	196.36	171.78
**EDSSMat62**	332.51	279.56	255.40	157.52	135.91
**EDSSMat70**	304.68	253.85	230.63	134.60	115.51
**EDSSMat75**	339.48	286.83	262.80	165.86	143.85
**EDSSMat80**	334.59	283.15	259.66	164.43	143.16
**EDSSMat90**	330.03	278.13	254.44	158.16	137.04

Supplementary Table S8. Z-score values for the comparison between five best performing search matrices (EDSSMat50, EDSSMat70, EDSSMat90, EDSSMat80 and EDSSMat60) and rest of *Standard, Disorder* and *EDSSMat* search matrices on highly disordered (HD) test dataset. Z-scores with |Z| ≥ 1.96 corresponds to > 95% confidence interval and hence significant. Non-significant Z-scores are highlighted in bold.

vs	**EDSSMat50**	**EDSSMat70**	**EDSSMat90**	**EDSSMat80**	**EDSSMat60**
**BLOSUM30**	52.52	48.50	48.29	47.28	47.25
**BLOSUM50**	108.47	104.49	104.68	104.28	104.35
**BLOSUM62**	136.14	132.27	132.63	132.51	132.63
**BLOSUM80**	114.71	110.83	111.04	110.68	110.77
**PAM120**	116.69	112.87	109.92	112.74	112.82
**PAM250**	73.24	69.23	66.01	68.38	68.39
**MD10**	214.62	210.83	211.74	212.47	212.74
**MD20**	194.06	190.26	190.98	191.44	191.66
**MD40**	156.33	152.42	152.94	153.07	153.24
**VTML10**	194.09	190.36	191.08	191.52	191.74
**VTML20**	181.38	177.69	178.29	178.58	178.77
**VTML40**	137.93	134.17	134.51	134.37	134.48
**VTML80**	109.67	105.82	105.99	105.58	105.65
**VTML120**	99.13	95.12	95.25	94.74	94.81
**VTML160**	105.33	101.51	101.65	101.18	101.24
**VTML200**	95.19	96.95	97.04	96.52	96.57
**DUNMat**	79.21	75.32	75.29	74.56	74.58
**MidicMat**	822.77	819.82	824.68	831.65	832.97
**Disorder40**	57.63	53.72	53.54	52.60	52.57
**Disorder60**	91.77	87.92	87.97	87.37	87.41
**Disorder85**	191.60	187.81	188.54	189.01	189.23
**EDSSMat50**	—	-4.07	-4.63	-6.21	-6.34
**EDSSMat60**	6.34	2.19	**1.66**	**0.11**	—
**EDSSMat62**	8.84	4.65	4.13	2.60	2.49
**EDSSMat70**	4.07	—	**-0.53**	-2.07	-2.19
**EDSSMat75**	7.52	3.44	2.93	**1.42**	**1.31**
**EDSSMat80**	6.21	2.07	1.5	—	**-0.11**
**EDSSMat90**	4.63	**0.53**	—	**-1.54**	**-1.66**

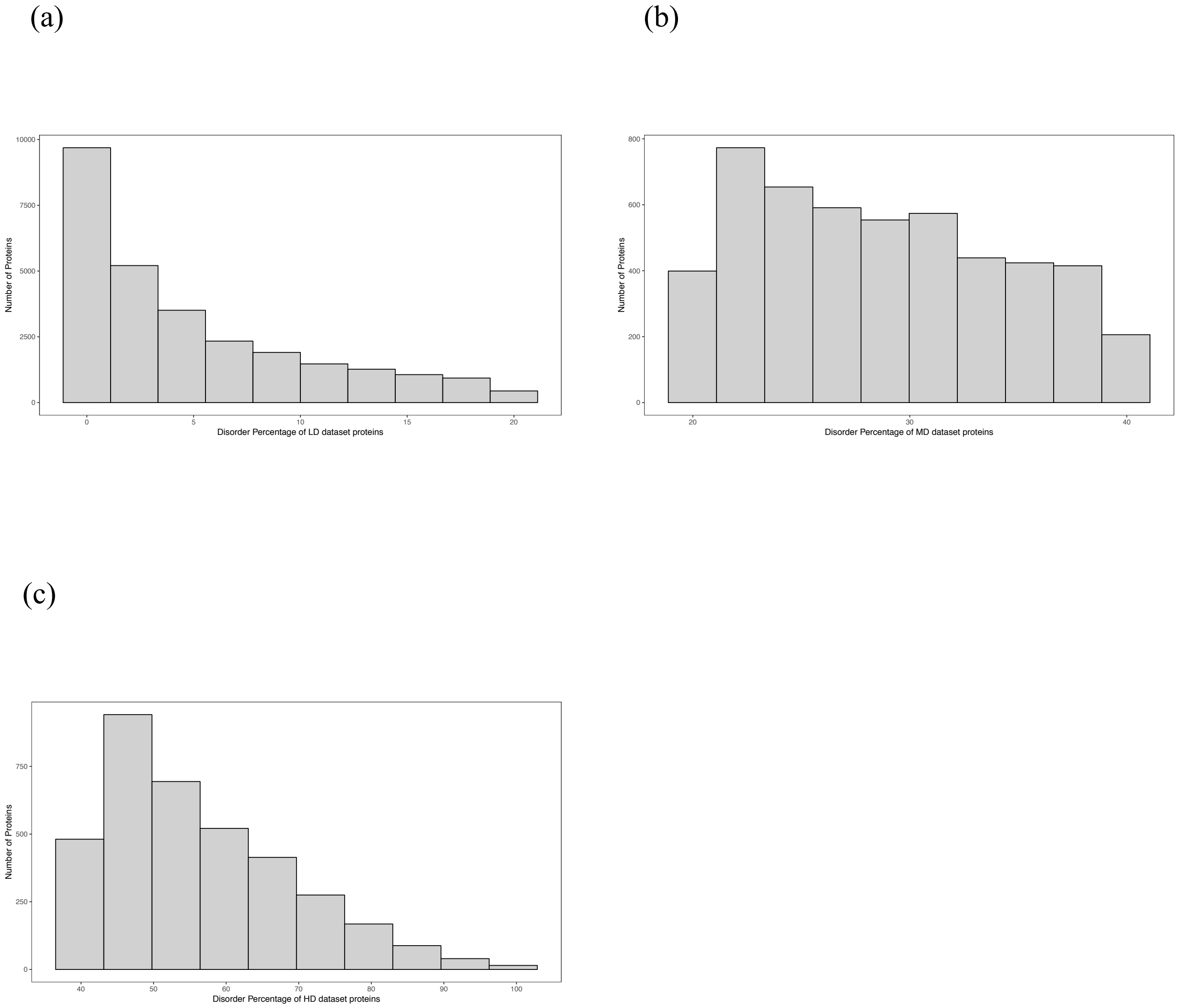

Supplementary Figure S1. Distribution of percent disorderedness among proteins of various test datasets: **(a)** Less Disordered (LD) ; **(b)** Moderately Disordered (MD) ; and **(c)** Highly Disordered (HD) is shown here.

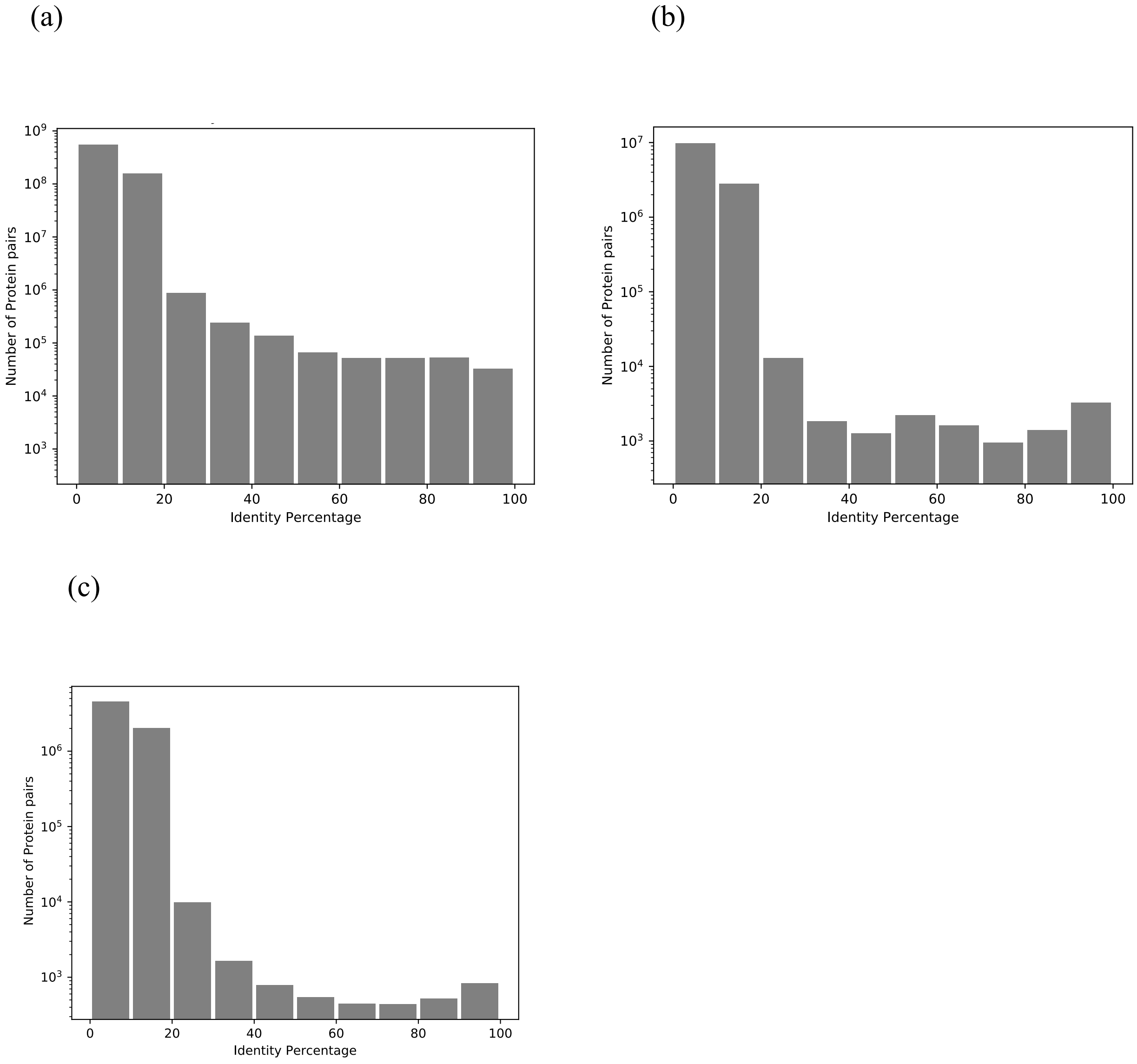

Supplementary Figure S2. Distribution of percent identities among proteins of various test datasets: **(a)** Less Disordered (LD) ; **(b)** Moderately Disordered (MD) ; and **(c)** Highly Disordered (HD) is shown here. X-axis represents identity percentage between a pair of sequences, and Y-axis denotes the number of protein sequence pairs for various identity percentages on logarithmic scale.

(a)

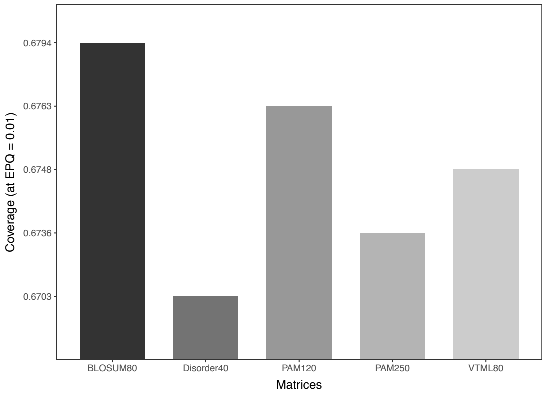

(b)

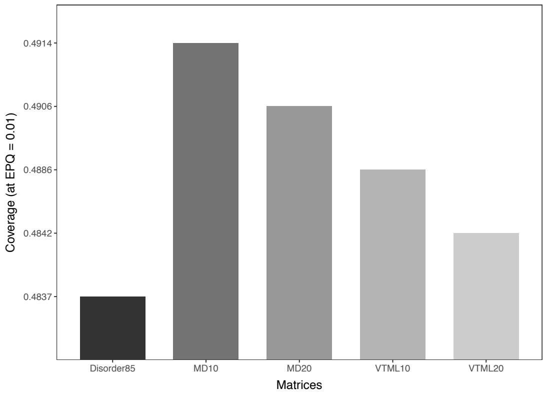

(c)

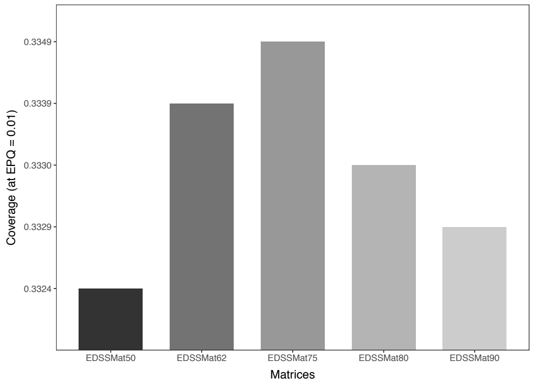

Supplementary Figure S3. Relative entropy-independent comparison of search matrices for homology detection using top 20 most populated protein families from all three test datasets: **(a)** Less Disordered (LD); **(b)** Moderately Disordered (MD); and **(c)** Highly Disordered (HD) test dataset. Quadratically normalised coverage measure (Q_{quad}) at 0.01 errors per query (EPQ) on y axis reports the fraction of true positive family relations at a restricted number of false positives. Height of a bar in the figure represents coverage (Q_{quad}) achieved by a matrix. All *EDSSMat* series of matrices achieved higher coverage values (Q_{quad}) than other comparing matrices on HD test dataset. On MD and LD test datasets, along with Disorder85, lower numbered MD and VTML search matrices are the best performers. Difference in coverage measures are also statistically significant as $Z \geq 1.96$ (Supplementary Table 6, 7 and 8).

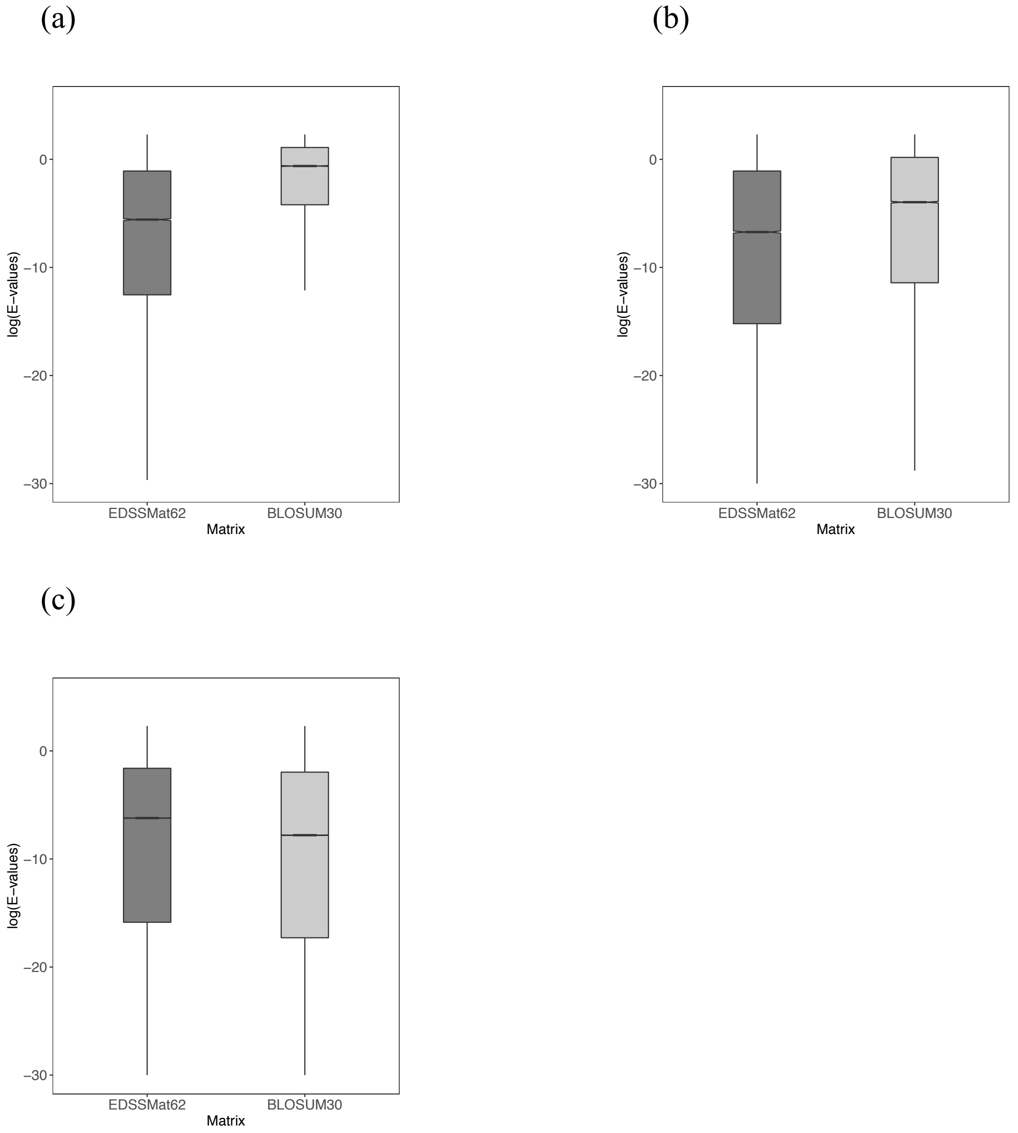

Supplementary Figure S4. Common homologs E-values distribution of BLOSUM and *EDSSMat* series of matrices. For representative purpose comparison of log10(E-values) distributions of common homologs of BLOSUM30 and EDSSMat62 on three different test datasets: **(a)** Highly Disordered (HD); **(b)** Moderately Disordered (MD); and **(c)** Less Disordered (LD) is shown here. EDSSMat62 matrix achieved lower E-values on test dataset comprised of highly disordered proteins i.e. HD test dataset, whereas BLOSUM30 attained lower E-values on LD test dataset enriched with ordered regions. Difference in E-values distributions for comparing matrices on all three test datasets are statistically significant (wilcoxon test p-value is $< 2.2e-16$).

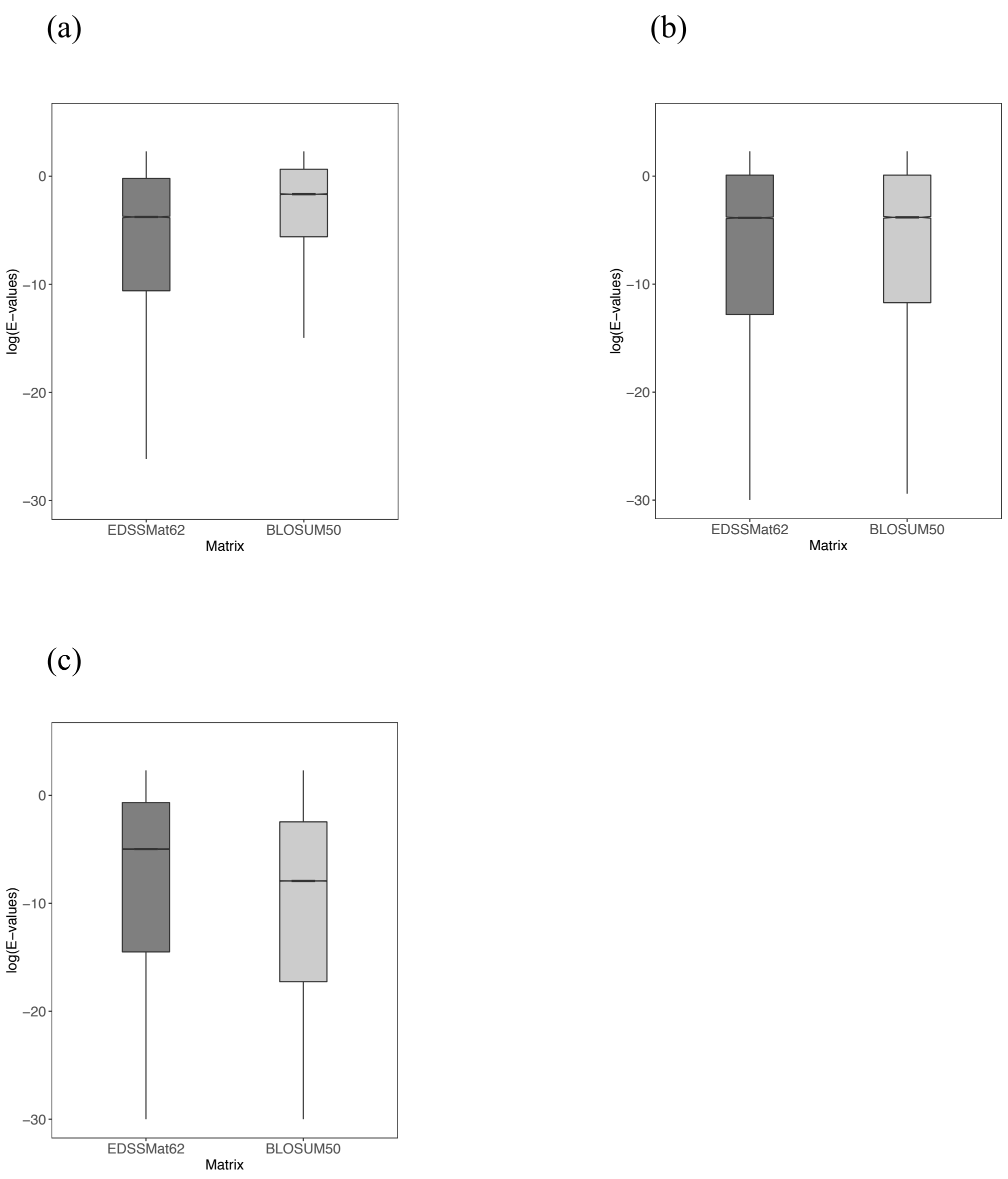

Supplementary Figure S5. Common homologs E-values distribution of BLOSUM and *EDSSMat* series of matrices. For representative purpose comparison of log10(E-values) distributions of common homologs of BLOSUM50 and EDSSMat62 on three different test datasets: **(a)** Highly Disordered (HD); **(b)** Moderately Disordered (MD); and **(c)** Less Disordered (LD) is shown here. EDSSMat62 matrix achieved lower E-values on test dataset comprised of highly disordered proteins i.e. HD test dataset, whereas BLOSUM50 attained lower E-values on LD test dataset enriched with ordered regions. Difference in E-values distributions for comparing matrices on all three test datasets are statistically significant (wilcoxon test p-value is $< 2.2e-16$).

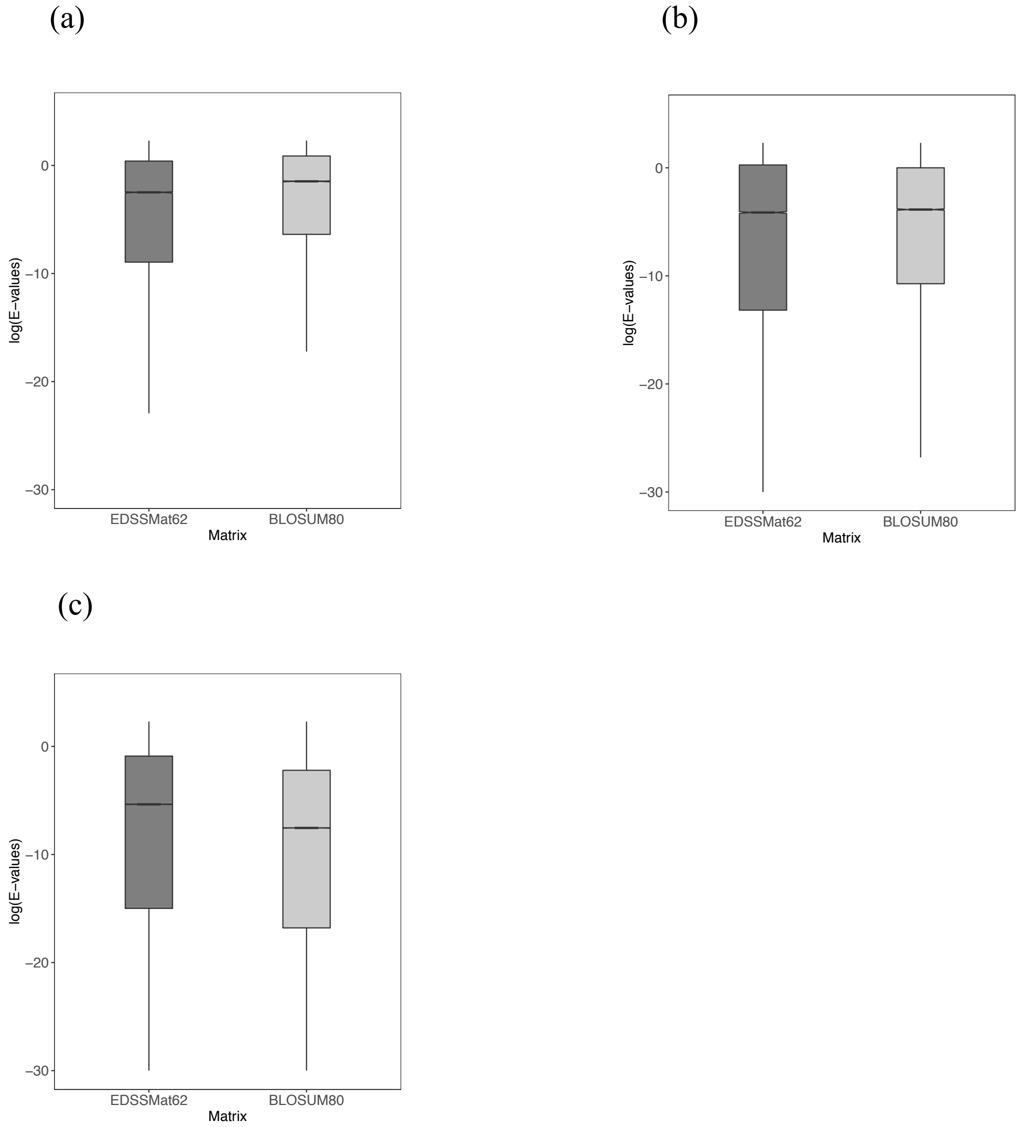

Supplementary Figure S6. Common homologs E-values distribution of BLOSUM and *EDSSMat* series of matrices. For representative purpose comparison of log10(E-values) distributions of common homologs of BLOSUM80 and EDSSMat62 on three different test datasets: **(a)** Highly Disordered (HD); **(b)** Moderately Disordered (MD); and **(c)** Less Disordered (LD) is shown here. EDSSMat62 matrix achieved lower E-values on test dataset comprised of highly disordered proteins i.e. HD test dataset, whereas BLOSUM80 attained lower E-values on LD test dataset enriched with ordered regions. Difference in E-values distributions for comparing matrices on all three test datasets are statistically significant (wilcoxon test p-value is $< 2.2e-16$).

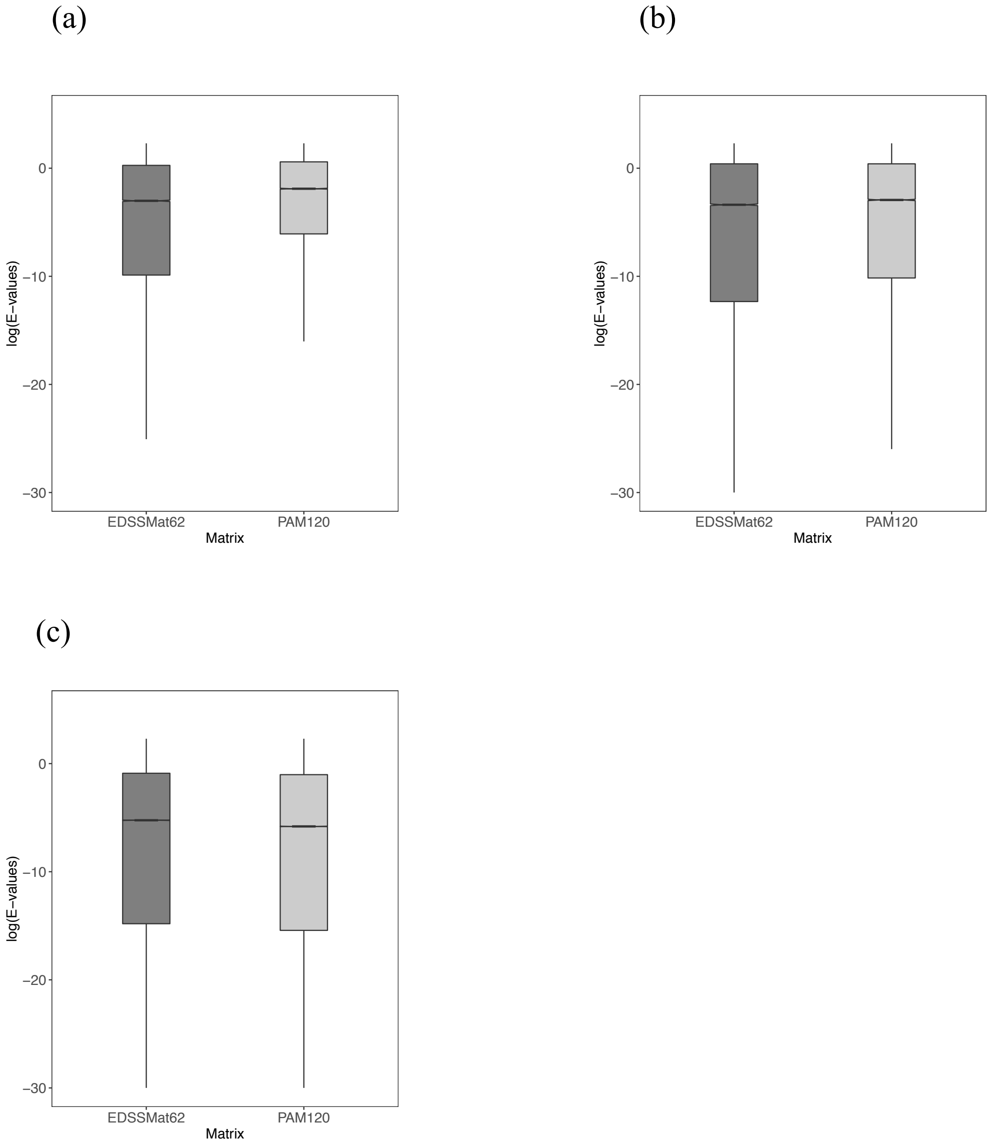

Supplementary Figure S7. Common homologs E-values distribution of PAM and *EDSSMat* series of matrices. For representative purpose comparison of log10(E-values) distributions of common homologs of PAM120 and EDSSMat62 on three different test datasets: **(a)** Highly Disordered (HD); **(b)** Moderately Disordered (MD); and **(c)** Less Disordered (LD) is shown here. EDSSMat62 matrix achieved lower E-values on test dataset comprised of highly disordered proteins i.e. HD test dataset, whereas PAM120 attained lower E-values on LD test dataset enriched with ordered regions. Difference in E-values distributions for comparing matrices on all three test datasets are statistically significant (wilcoxon test p-value is < 2.2e-16).

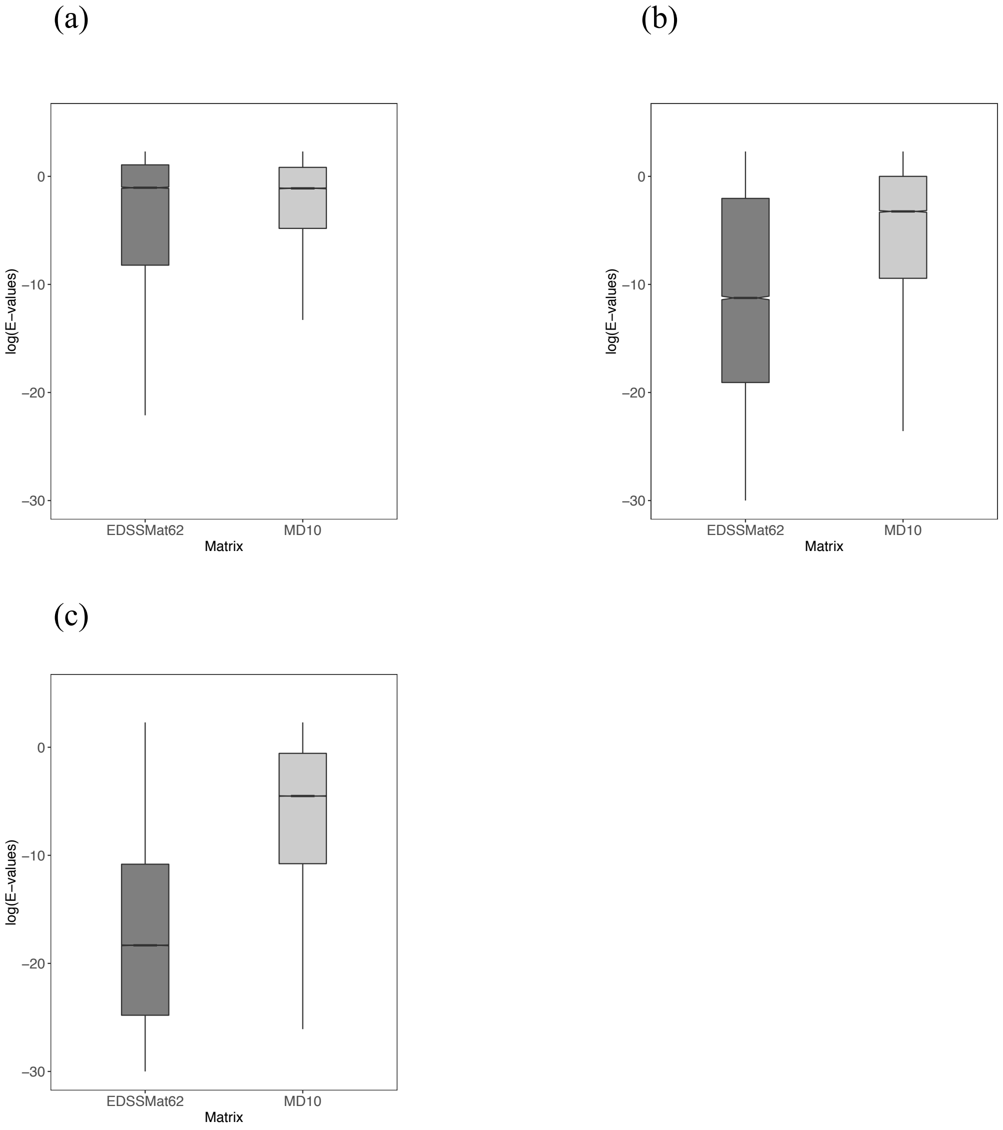

Supplementary Figure S8. Common homologs E-values distribution of MD and *EDSSMat* series of matrices. For representative purpose comparison of log10(E-values) distributions of common homologs of MD10 and EDSSMat62 on three different test datasets: **(a)** Highly Disordered (HD); **(b)** Moderately Disordered (MD); and **(c)** Less Disordered (LD) is shown here. EDSSMat62 matrix achieved lower E-values on MD and LD test datasets, whereas MD10 attained marginally lower E-values than EDSSMat62 on test dataset highly enriched with disordered regions i.e. HD test dataset. Difference in E-values distributions for comparing matrices on all three test datasets are statistically significant (wilcoxon test p-value is $< 2.2e-16$).

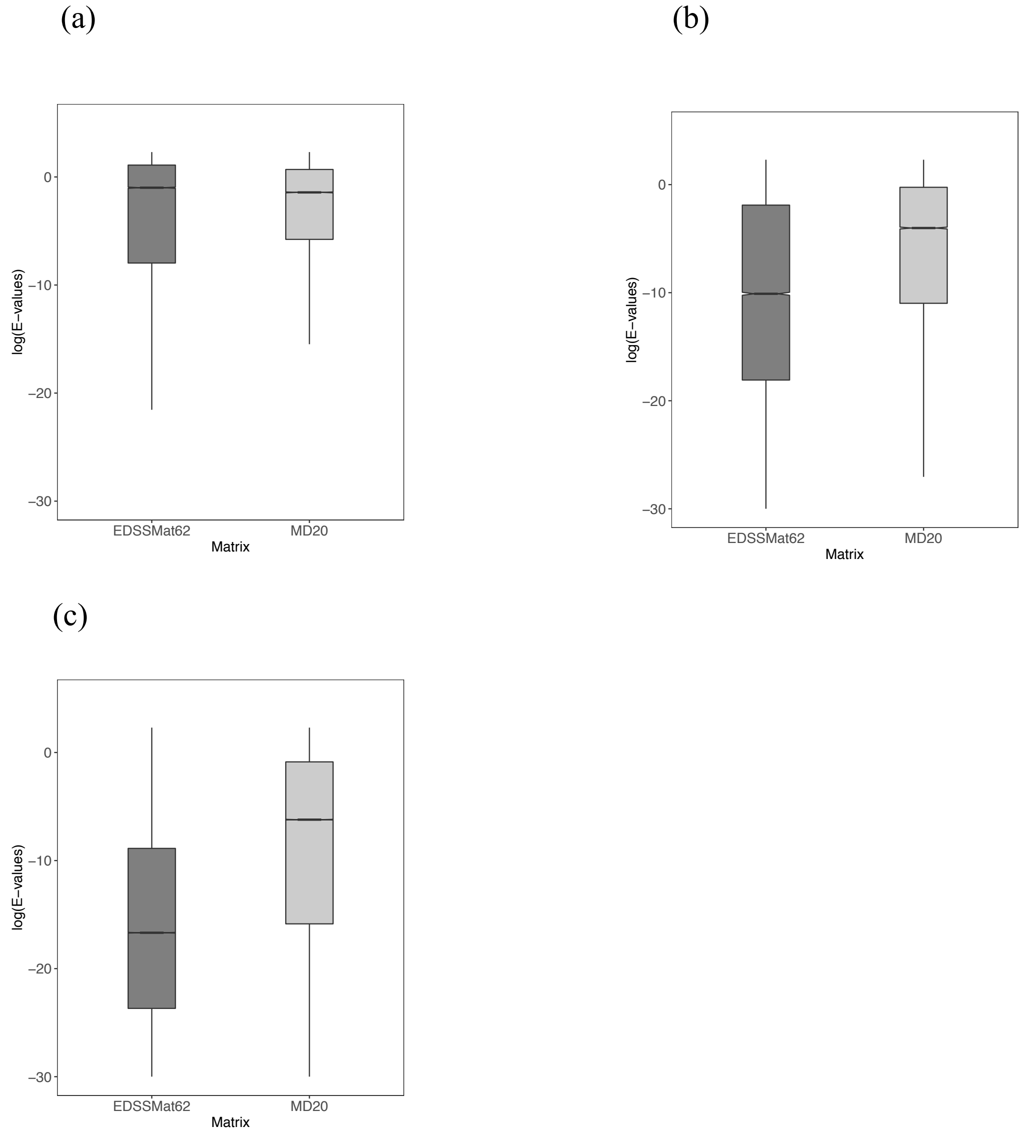

Supplementary Figure S9. Common homologs E-values distribution of MD and *EDSSMat* series of matrices. For representative purpose comparison of log10(E-values) distributions of common homologs of MD20 and EDSSMat62 on three different test datasets: **(a)** Highly Disordered (HD); **(b)** Moderately Disordered (MD); and **(c)** Less Disordered (LD) is shown here. EDSSMat62 matrix achieved lower E-values on MD and LD test datasets, whereas MD20 attained marginally lower E-values than EDSSMat62 on test dataset highly enriched with disordered regions i.e. HD test dataset. Difference in E-values distributions for comparing matrices on all three test datasets are statistically significant (HD and LD test dataset: wilcoxon test p-value is < 2.2e-16; MD test dataset: wilcoxon test p-value = 0.0040).

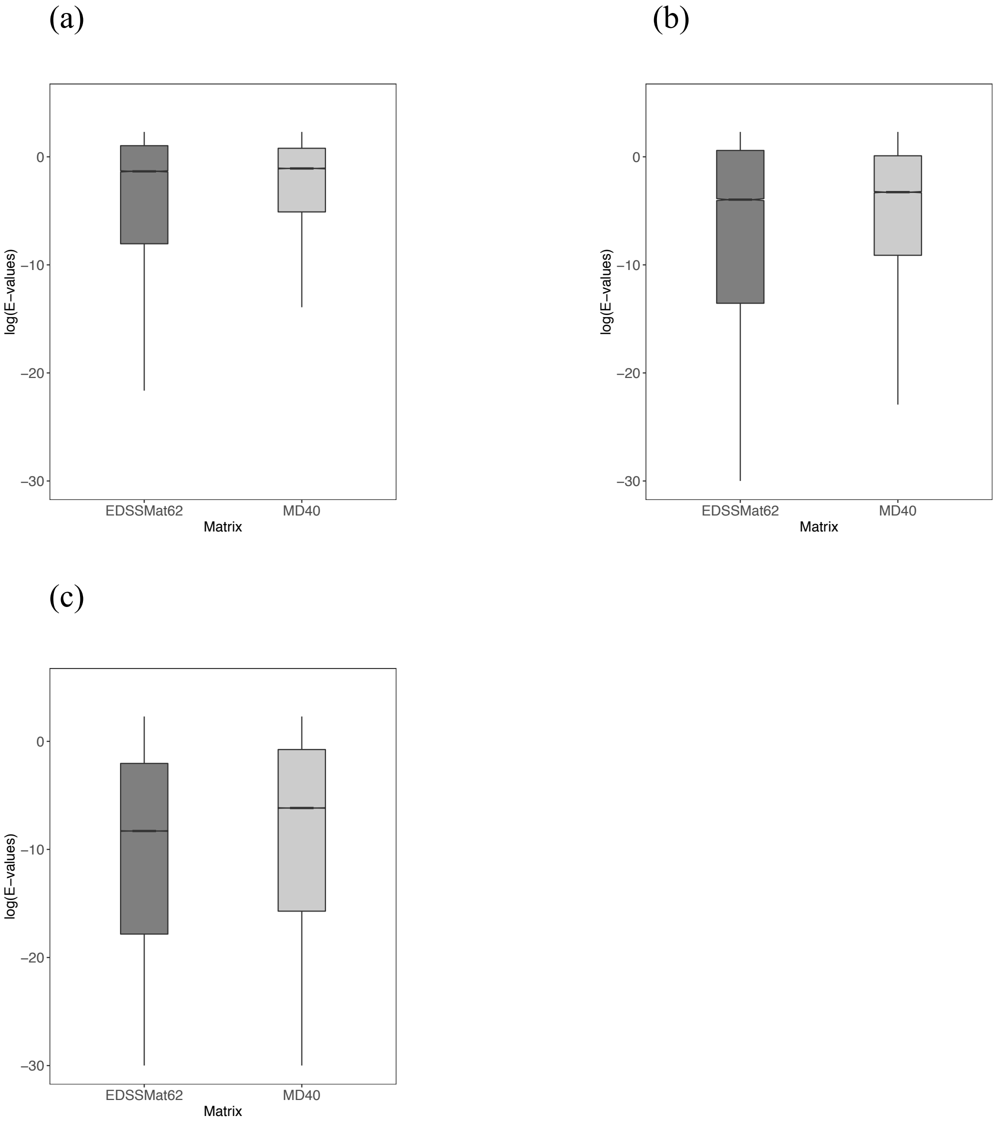

Supplementary Figure S10. Common homologs E-values distribution of MD and *EDSSMat* series of matrices. For representative purpose comparison of log10(E-values) distributions of common homologs of MD40 and EDSSMat62 on three different test datasets: **(a)** Highly Disordered (HD); **(b)** Moderately Disordered (MD); and **(c)** Less Disordered (LD) is shown here. EDSSMat62 matrix achieved lower E-values on all three test datasets. Difference in E-values distributions for comparing matrices on all three test datasets are statistically significant (wilcoxon test p-value is < 2.2e-16).

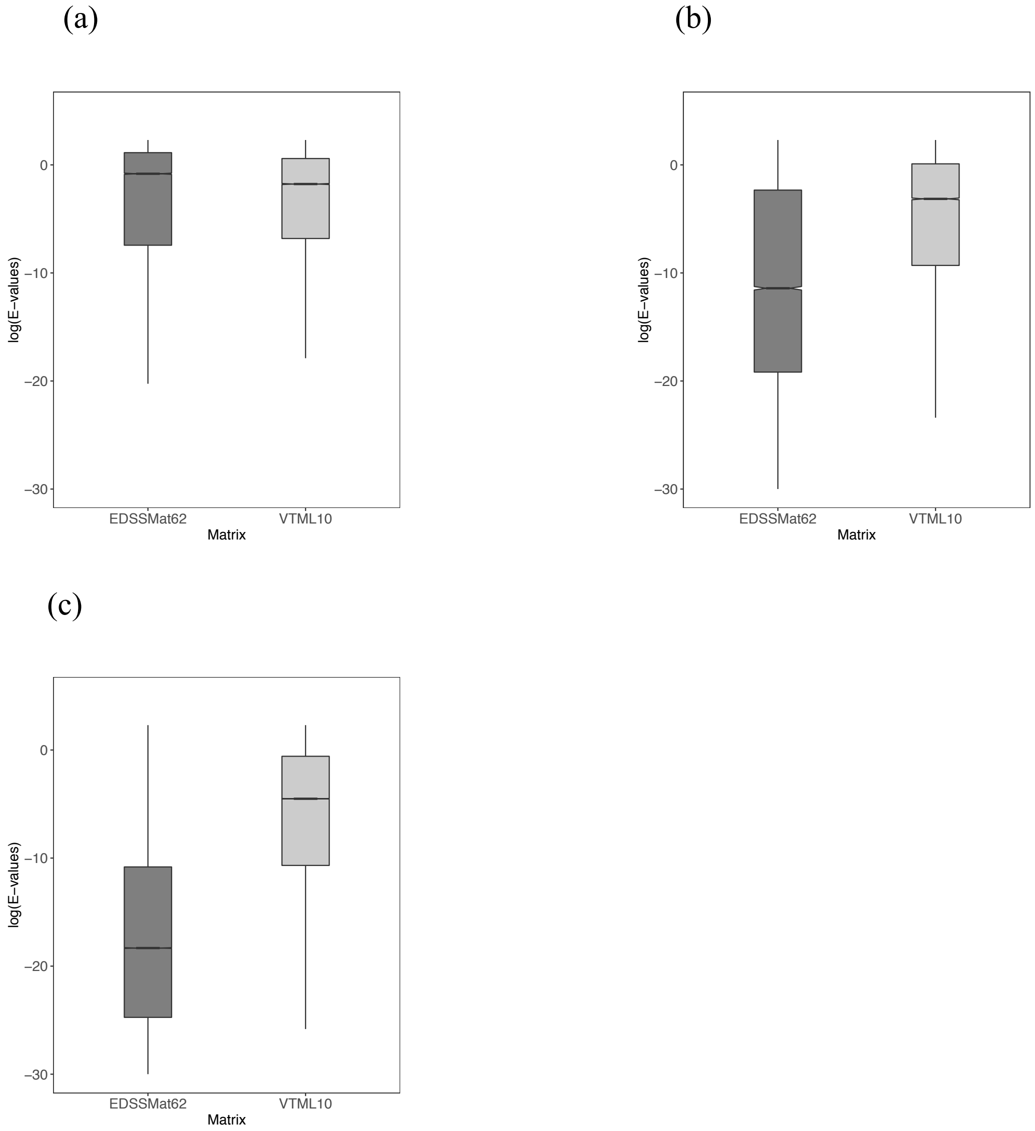

Supplementary Figure S11. Common homologs E-values distribution of VTML and *EDSSMat* series of matrices. For representative purpose comparison of log10(E-values) distributions of common homologs of VTML10 and EDSSMat62 on three different test datasets: **(a)** Highly Disordered (HD); **(b)** Moderately Disordered (MD); and **(c)** Less Disordered (LD) is shown here. EDSSMat62 matrix achieved lower E-values on MD and LD test datasets, whereas VTML10 attained marginally lower E-values on test dataset highly enriched with disordered regions i.e. HD test dataset. Difference in E-values distributions for comparing matrices on all three test datasets are statistically significant (MD and LD test datasets: wilcoxon test p-value is $< 2.2e-16$; HD test dataset: wilcoxon test p-value = 0.0011).

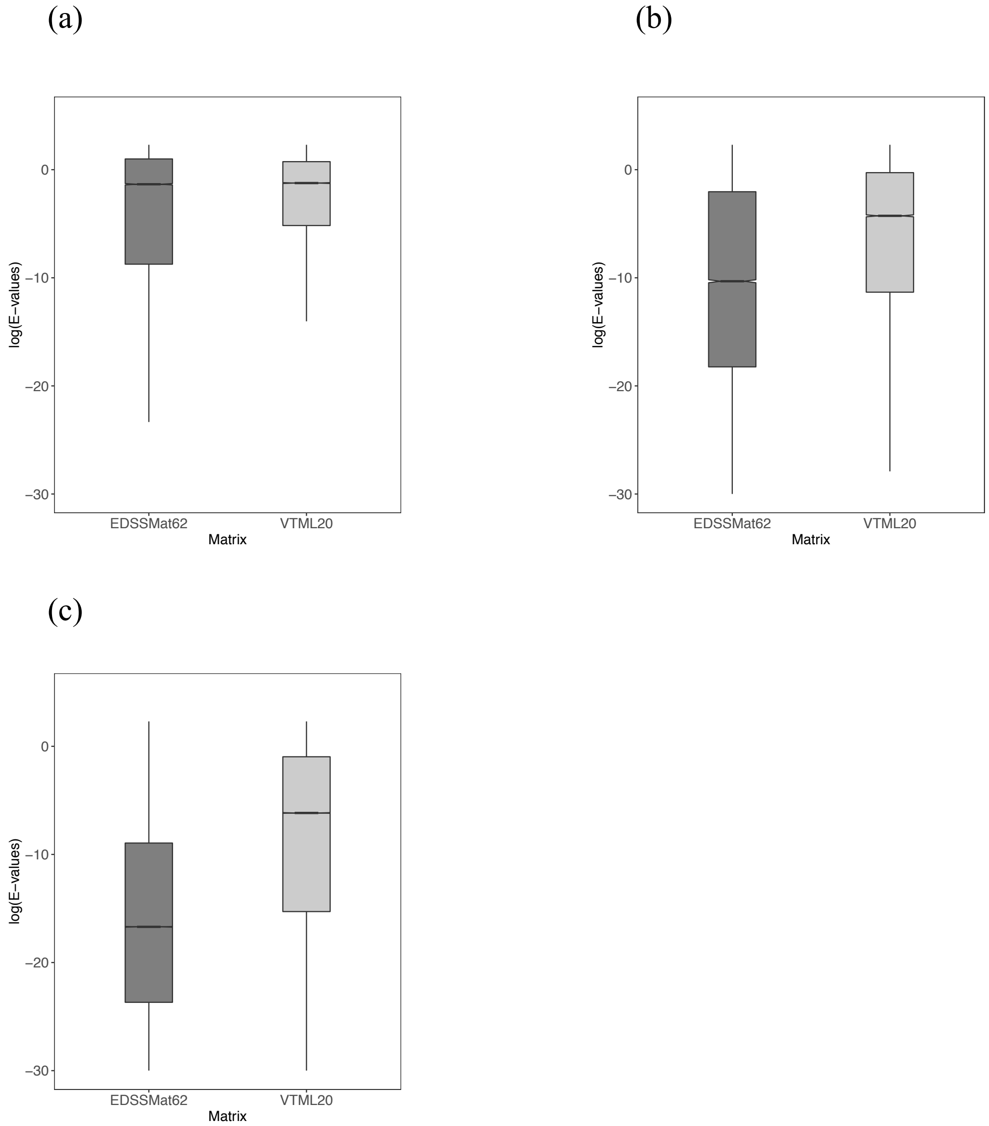

Supplementary Figure S12. Common homologs E-values distribution of VTML and *EDSSMat* series of matrices. For representative purpose comparison of log10(E-values) distributions of common homologs of VTML20 and EDSSMat62 on three different test datasets: **(a)** Highly Disordered (HD); **(b)** Moderately Disordered (MD); and **(c)** Less Disordered (LD) is shown here. EDSSMat62 matrix achieved lower E-values on all three test datasets. Difference in E-values distributions for comparing matrices on all three test datasets are statistically significant (wilcoxon test p-value is < 2.2e-16).

(a)

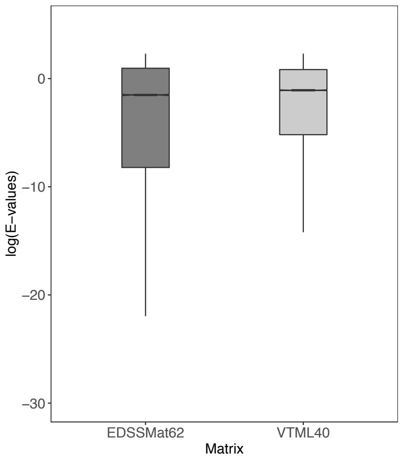

(b)

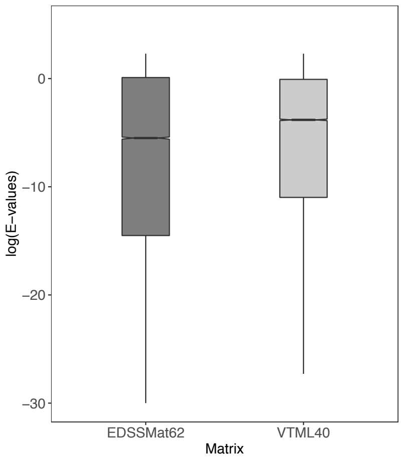

(c)

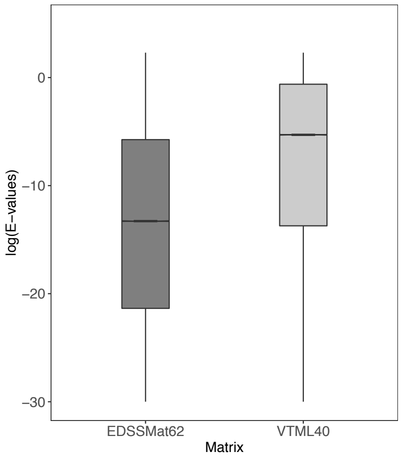

Supplementary Figure S13. Common homologs E-values distribution of VTML and *EDSSMat* series of matrices. For representative purpose comparison of log10(E-values) distributions of common homologs of VTML40 and EDSSMat62 on three different test datasets: **(a)** Highly Disordered (HD); **(b)** Moderately Disordered (MD); and **(c)** Less Disordered (LD) is shown here. EDSSMat62 matrix achieved lower E-values on all the three test datasets. Difference in E-values distributions for comparing matrices on all three test datasets are statistically significant (wilcoxon test p-value is $< 2.2e-16$).

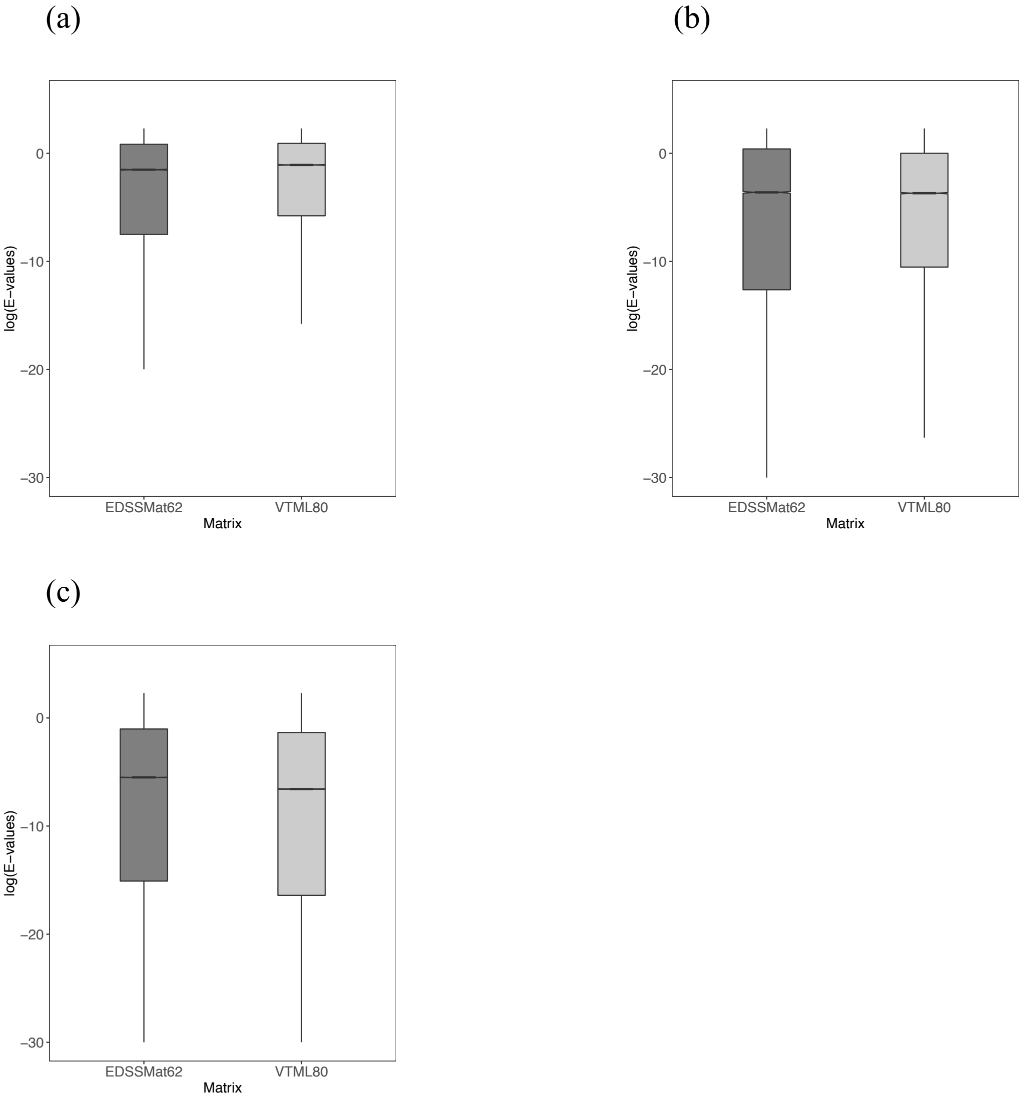

Supplementary Figure S14. Common homologs E-values distribution of VTML and *EDSSMat* series of matrices. For representative purpose comparison of log10(E-values) distributions of common homologs of VTML80 and EDSSMat62 on three different test datasets: **(a)** Highly Disordered (HD); **(b)** Moderately Disordered (MD); and **(c)** Less Disordered (LD) is shown here. EDSSMat62 matrix achieved lower E-values on test dataset comprised of highly disordered proteins i.e. HD test dataset, whereas VTML80 attained lower E-values on LD test dataset enriched with ordered regions. Difference in E-values distributions for comparing matrices on all three test datasets are statistically significant (wilcoxon test p-value is $< 2.2e-16$).

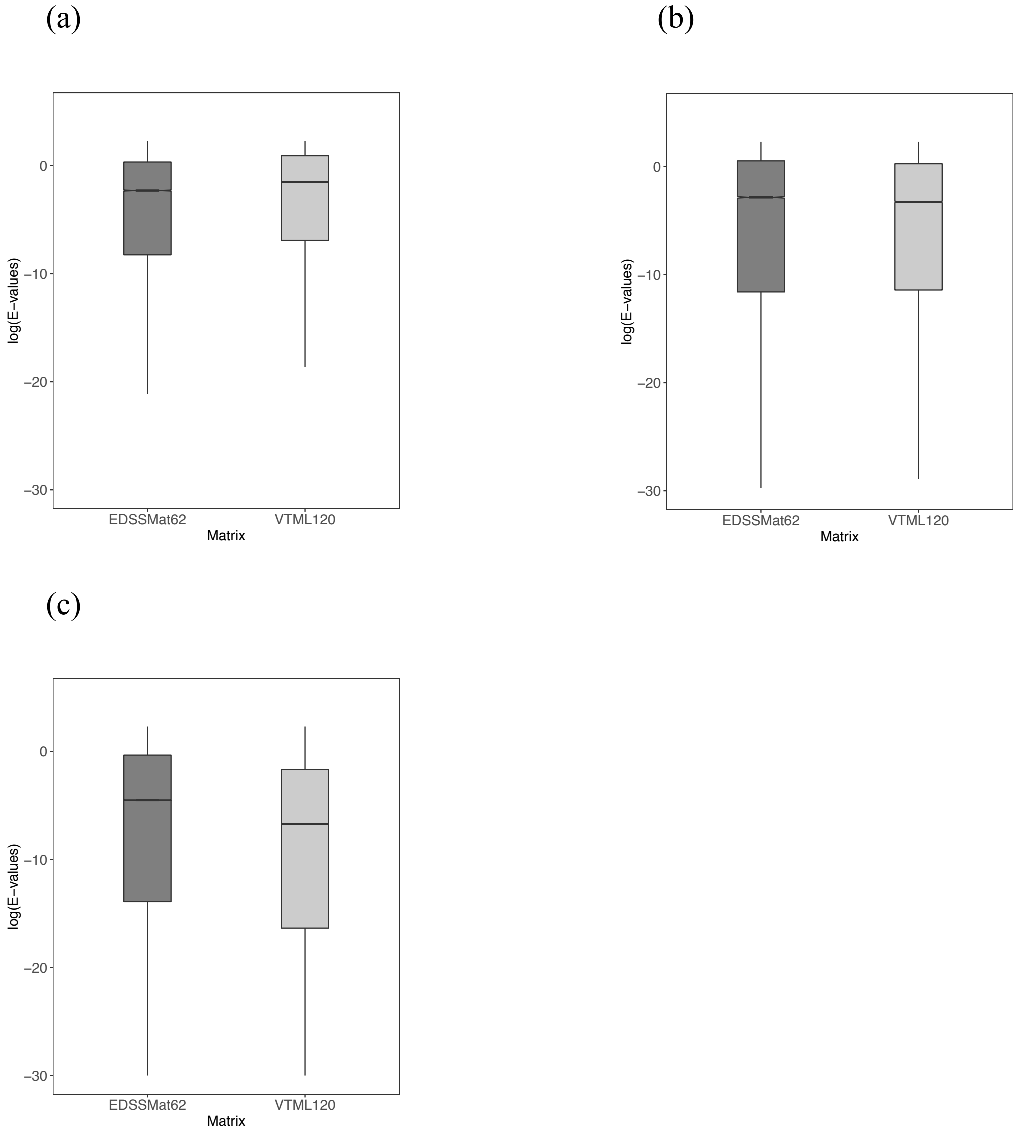

Supplementary Figure S15. Common homologs E-values distribution of VTML and *EDSSMat* series of matrices. For representative purpose comparison of log10(E-values) distributions of common homologs of VTML120 and EDSSMat62 on three different test datasets: **(a)** Highly Disordered (HD); **(b)** Moderately Disordered (MD); and **(c)** Less Disordered (LD) is shown here. EDSSMat62 matrix achieved lower E-values on test dataset comprised of highly disordered proteins i.e. HD test dataset, whereas VTML120 attained lower E-values on LD test dataset enriched with ordered regions. Difference in E-values distributions for comparing matrices are statistically significant on HD and LD test datasets (wilcoxon test p-value is < 2.2e-16), and insignificant on MD test dataset (wilcoxon test p-value = 0.1358).

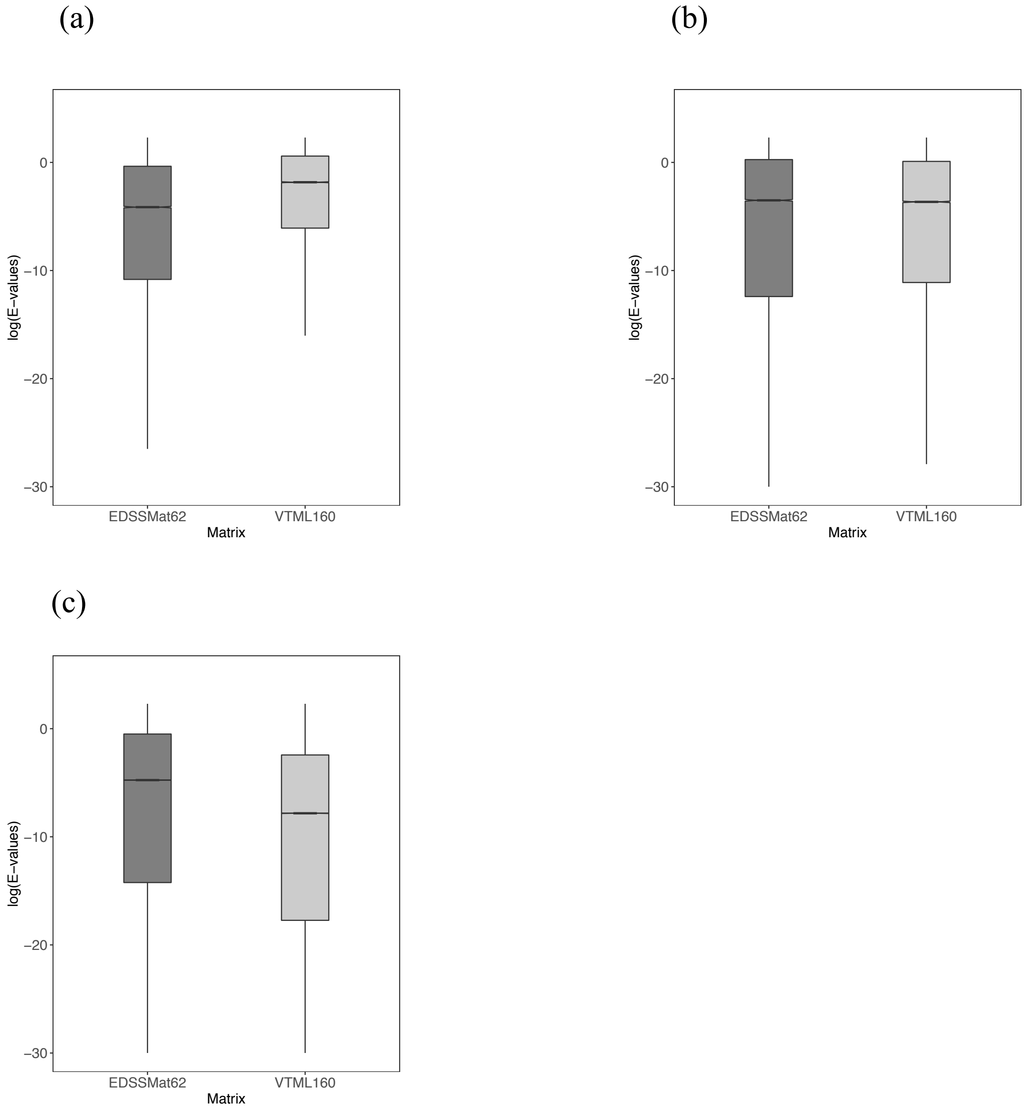

Supplementary Figure S16. Common homologs E-values distribution of VTML and *EDSSMat* series of matrices. For representative purpose comparison of log10(E-values) distributions of common homologs of VTML160 and EDSSMat62 on three different test datasets: **(a)** Highly Disordered (HD); **(b)** Moderately Disordered (MD); and **(c)** Less Disordered (LD) is shown here. EDSSMat62 matrix achieved lower E-values on test dataset comprised of highly disordered proteins i.e. HD test dataset, whereas VTML160 attained lower E-values on LD test dataset enriched with ordered regions. Difference in E-values distributions for comparing matrices on all three test datasets are statistically significant (wilcoxon test p-value is $< 2.2e-16$).

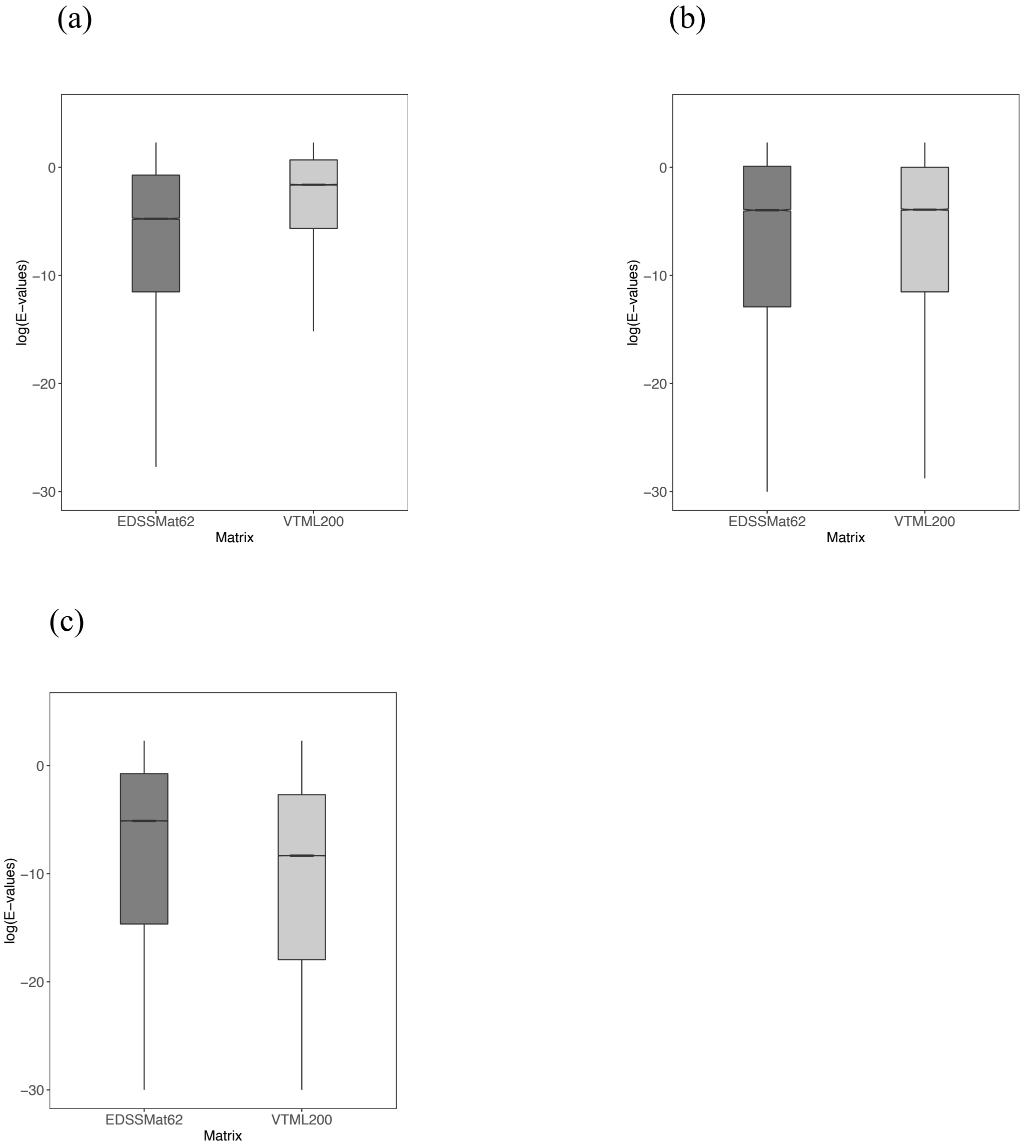

Supplementary Figure S17. Common homologs E-values distribution of VTML and *EDSSMat* series of matrices. For representative purpose comparison of log10(E-values) distributions of common homologs of VTML200 and EDSSMat62 on three different test datasets: **(a)** Highly Disordered (HD); **(b)** Moderately Disordered (MD); and **(c)** Less Disordered (LD) is shown here. EDSSMat62 matrix achieved lower E-values on test dataset comprised of highly disordered proteins i.e. HD test dataset, whereas VTML200 attained lower E-values on LD test dataset enriched with ordered regions. Difference in E-values distributions for all pair of comparing matrices are statistically significant (wilcoxon test p-value is $< 2.2e-16$).

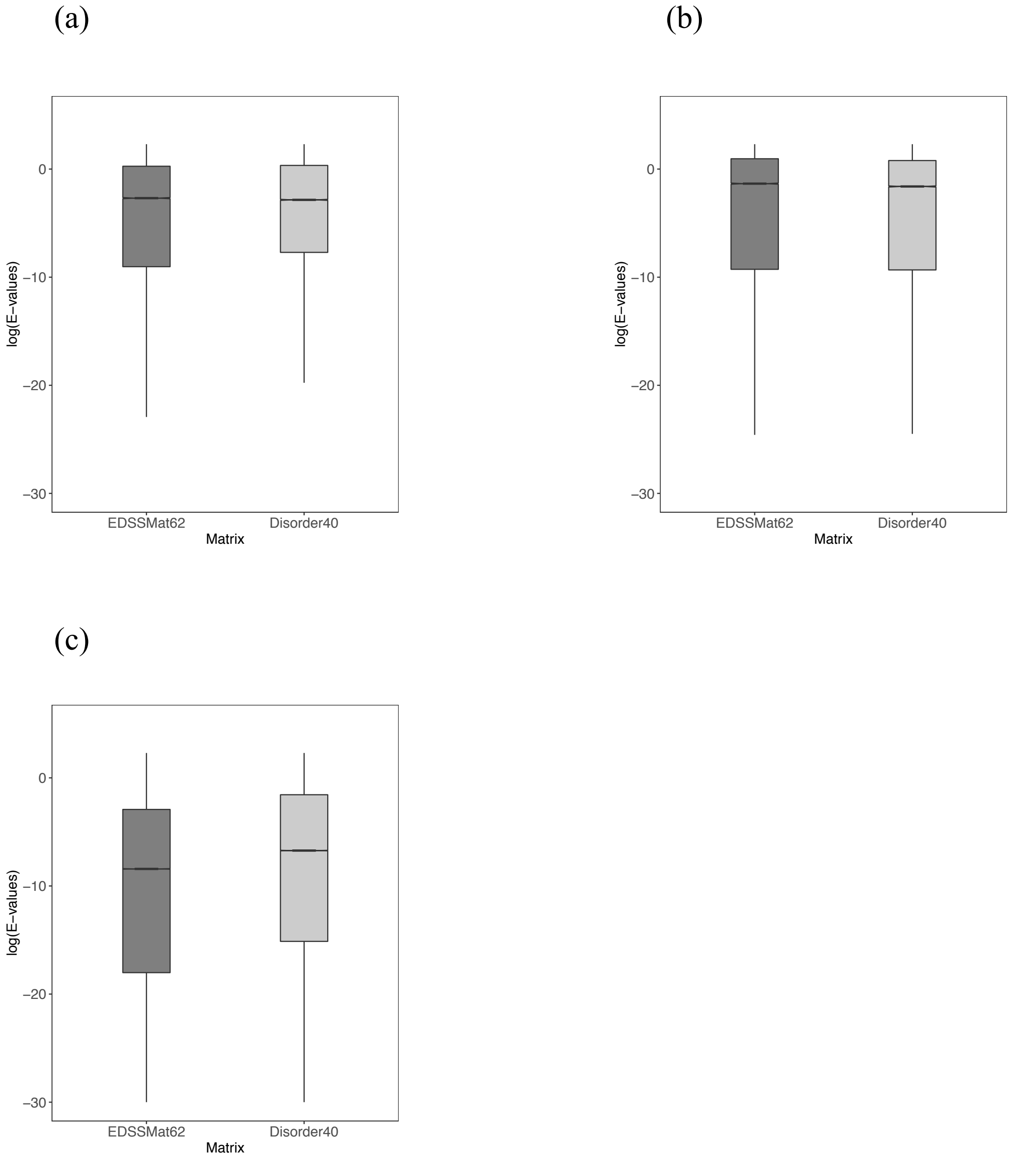

Supplementary Figure S18. Common homologs E-values distribution of Disorder and *EDSSMat* series of matrices. For representative purpose comparison of log10(E-values) distributions of common homologs of Disorder40 and EDSSMat62 on three different test datasets: **(a)** Highly Disordered (HD); **(b)** Moderately Disordered (MD); and **(c)** Less Disordered (LD) is shown here. Disorder40 matrix achieved lower E-values on test dataset comprised of highly disordered proteins i.e. HD test dataset, whereas EDSSMat62 attained lower E-values on dataset enriched with ordered regions. Difference in E-values distributions for comparing matrices on all three test datasets are statistically significant (HD and LD test datasets: wilcoxon test p-value is < 2.2e-16; MD test dataset wilcoxon test p-value = 6.803e -10).

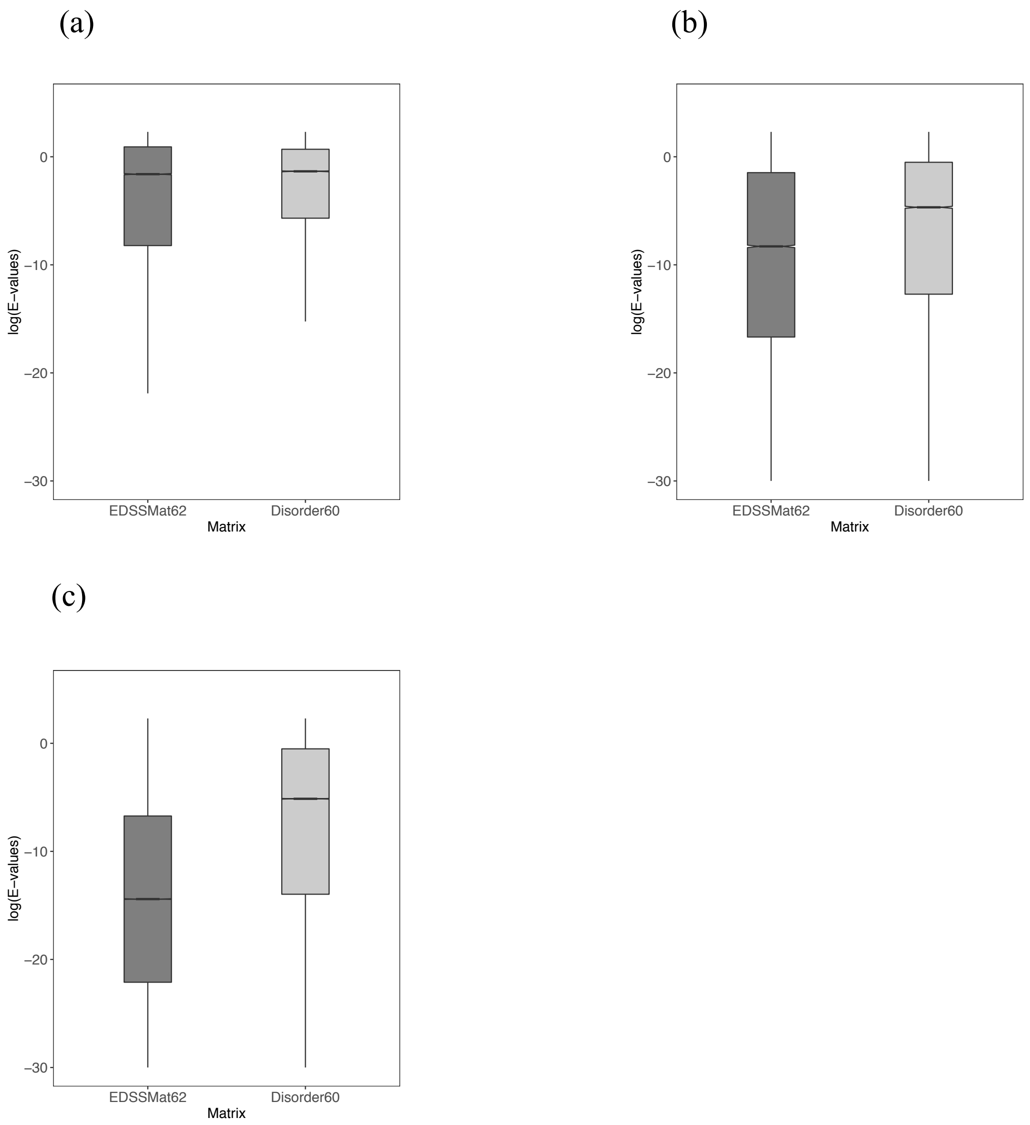

Supplementary Figure S19. Common homologs E-values distribution of Disorder and *EDSSMat* series of matrices. For representative purpose comparison of log10(E-values) distributions of common homologs of Disorder60 and EDSSMat62 on three different test datasets: **(a)** Highly Disordered (HD); **(b)** Moderately Disordered (MD); and **(c)** Less Disordered (LD) is shown here. EDSSMat62 matrix achieved lower E-values on all three test datasets. Difference in E-values distributions for comparing matrices are statistically significant on HD and LD test datasets (wilcoxon test p-value is $< 2.2e\text{-}16$), and insignificant on MD test dataset (wilcoxon test p-value = 0.3705).

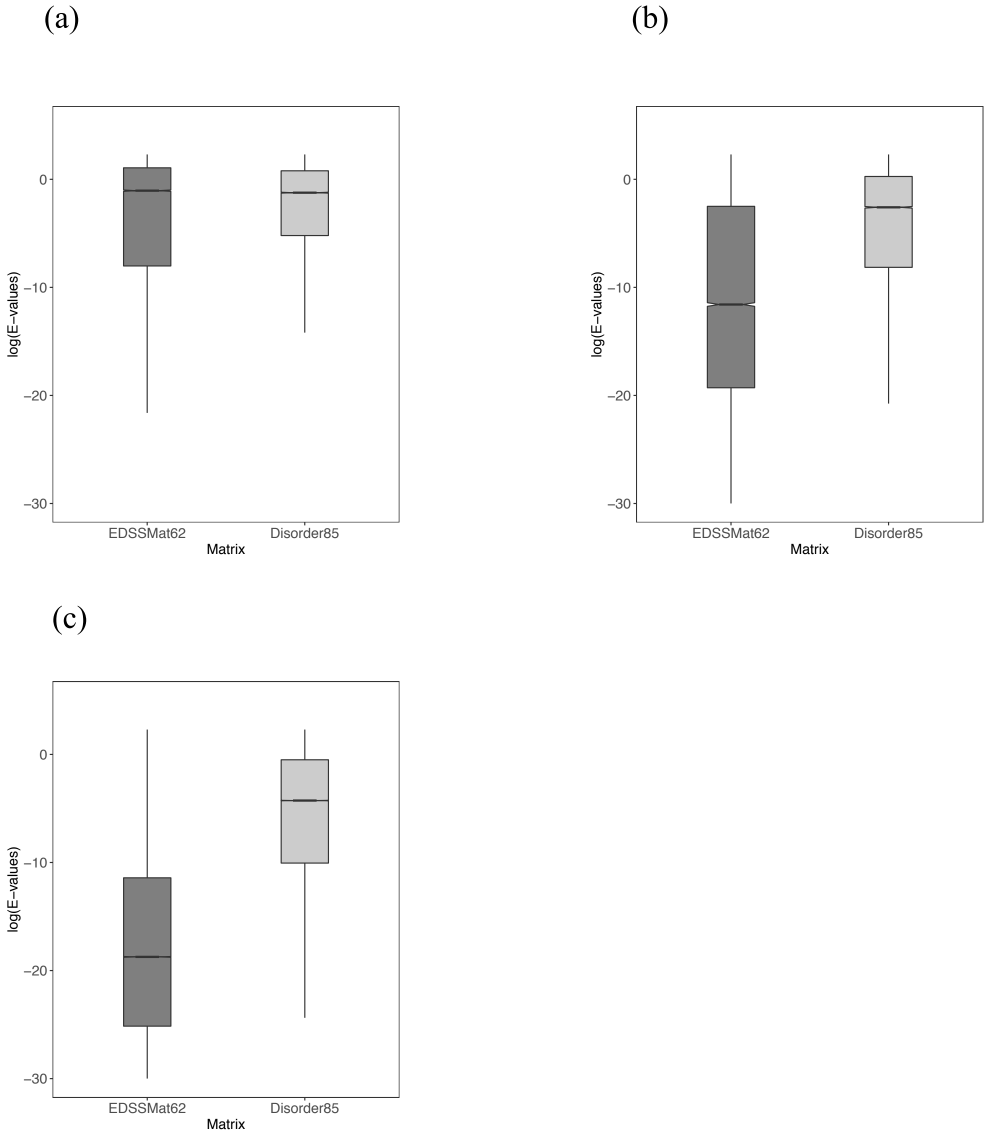

Supplementary Figure S20. Common homologs E-values distribution of Disorder and *EDSSMat* series of matrices. For representative purpose comparison of log10(E-values) distributions of common homologs of Disorder85 and EDSSMat62 on three different test datasets: **(a)** Highly Disordered (HD); **(b)** Moderately Disordered (MD); and **(c)** Less Disordered (LD) is shown here. EDSSMat62 matrix achieved lower E-values on MD and LD test datasets, whereas Disorder85 attained marginally lower E-values on test dataset highly enriched with disordered regions i.e. HD test dataset. Difference in E-values distributions for comparing matrices on all three test datasets are statistically significant (wilcoxon test p-value is $< 2.2e-16$).

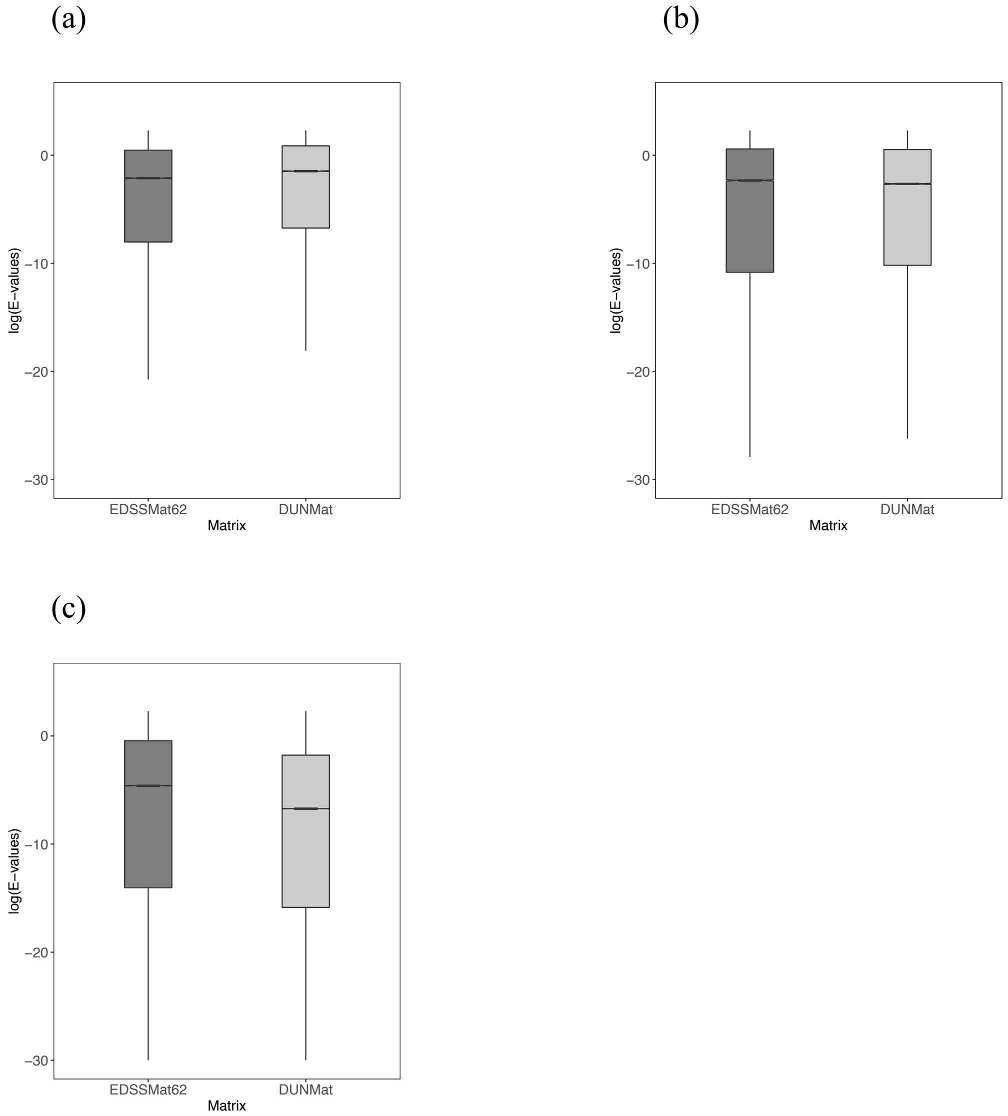

Supplementary Figure S21. Common homologs E-values distribution of DUNMat and *EDSSMat* series of matrices. For representative purpose comparison of log10(E-values) distributions of common homologs of DUNMat and EDSSMat62 on three different test datasets: **(a)** Highly Disordered (HD); **(b)** Moderately Disordered (MD); and **(c)** Less Disordered (LD) is shown here. EDSSMat62 matrix achieved lower E-values on test dataset comprised of highly disordered proteins i.e. HD test dataset, whereas DUNMat attained lower E-values on LD test dataset enriched with ordered regions. Difference in E-values distributions for comparing matrices on all three test datasets are statistically significant (wilcoxon test p-value is < 2.2e-16).
